# A comprehensive analysis of gasdermin family gene as therapeutic targets in pan-cancer

**DOI:** 10.1038/s41598-022-17100-7

**Published:** 2022-08-03

**Authors:** Cheng-Long Huo, Yan Deng, Zhen-Gang Sun

**Affiliations:** Department of Hepatobiliary Surgery, Jing Zhou Hospital Affiliated Yangtze University, Jing Zhou, 434020 Hubei China

**Keywords:** Cancer, Computational biology and bioinformatics, Drug discovery, Genetics, Immunology, Biomarkers, Oncology, Risk factors

## Abstract

Six members of the gasdermin family are involved in various biological functions in malignant tumors. The present study aimed to perform a comprehensive analysis of gasdermin family genes in pan-cancer. Raw data was acquired from the genotype-tissue expression (GTEx) and the Cancer Genome Atlas. High inter-tumor heterogeneity in the expression between paracancerous and tumor tissues was observed across cancers. Survival analysis confirmed that the risk or protective effects of gasdermin family members on prognosis depended on the cancer types. The mutation frequency appeared to be high, and the mutation group had a worse prognosis. Besides, gasdermin family genes were associated with immune infiltrate subtypes, stromal and immune cell infiltration levels, TMB, MSI, immune checkpoint gene expression, and tumor stemness scores. Moreover, gasdermin family gene expressions affected the expressions of MMR genes and methyltransferases and could predict cancer cells sensitivity to chemotherapeutic drugs. Subsequently, the findings were double-checked in LIHC and PAAD. GSEA results indicated the gasdermin family genes mainly involved in tumor metabolism and immune microenvironment remodeling related signaling pathways. In conclusion, our findings confirmed that gasdermin family genes were potential therapeutic cancer targets in pan-cancer.

## Introduction

Cancer has evolved into a devastating disease that jeopardizes public health^1^. According to the World Health Organization (WHO) estimates, the number of global cancer cases may increase by 60% in the next 20 years, with cancer mortality rapidly increasing^2^. Although tremendous efforts have been devoted to early diagnosis and the adoption of innovative approaches, including immune checkpoint blocking therapy and targeted therapy, the survival benefit remains limited^3–5^. Hence, cancer remains a major threat to human health^6^. Undoubtedly, tumorigenesis and cancer progression are fueled by gene mutations and abnormal proteins^7^. Moreover, the application of genomic technologies in tumors allows for targeted therapy and immunotherapy to be effective in some cancers^8^. However, only a few specific tumors have targetable molecules. Hence, reliable cancer biomarkers and prospective targets are needed to guide individualized therapy.

Gasdermin family comprises conserved N-terminal and C-terminal portions in humans. Based on the homology, several gasdermin family members have been identified, and the family currently includes six paralogous genes: gasdermin A (GSDMA), gasdermin B (GSDMB), gasdermin C (GSDMC), gasdermin D (GSDMD), gasdermin E (GSDME), and pejvakin (PJVK)^9,10^. The gasdermin family has been involved in the inflammatory response, cell-growth regulation, and host defense^9,11,12^. Previous researches showed that GSDMA, GSDMC, GSDMD, GSDME, and PJVK mainly functioned as anti-oncogenes, while GSDMB acted as an oncogene in certain cancers^13,14^. However, the prognostic significance of each family gene varies in different malignancies. Previous studies confirmed that over-expression of GSDMC enhanced cell proliferation and xenograft tumor growth^15,16^. What is more, as the executors of pyroptosis, these genes’ N-terminal domains exhibit pore-forming activity on the cell membrane, which functions as a double-edged sword in tumor pathogenesis^17–20^. These findings implied that they could be exploited as cancer therapy targets^21,22^.

The current study conducted a comprehensive analysis to investigate gasdermin family gene’ differential expressions between tumor and normal tissues and the prognostic value in pan-cancer. Moreover, we comprehensively looked at the potential correlations between gasdermin family genes expression and the genetic alterations, tumor microenvironment, immunological subtypes, immune checkpoints biomarkers, immune neoantigens, tumor mutational burden (TMB), microsatellite instability (MSI), and the expression of mismatch repair (MMR) genes and DNA methyltransferases across various tumor types. Furthermore, we also implored the associations of gasdermin family gene expression with tumor stemness score and treatment sensitivity in pan-cancer. Subsequently, the potential functions of the gasdermin family genes were comprehensively assessed in LIHC and PAAD. Finally, gene set enrichment analysis (GSEA) was performed to investigate the special functions and mechanisms of the gasdermin family in cancers.

## Results

### Expression profiles of gasdermin family genes in normal tissues

**Figure 1:**
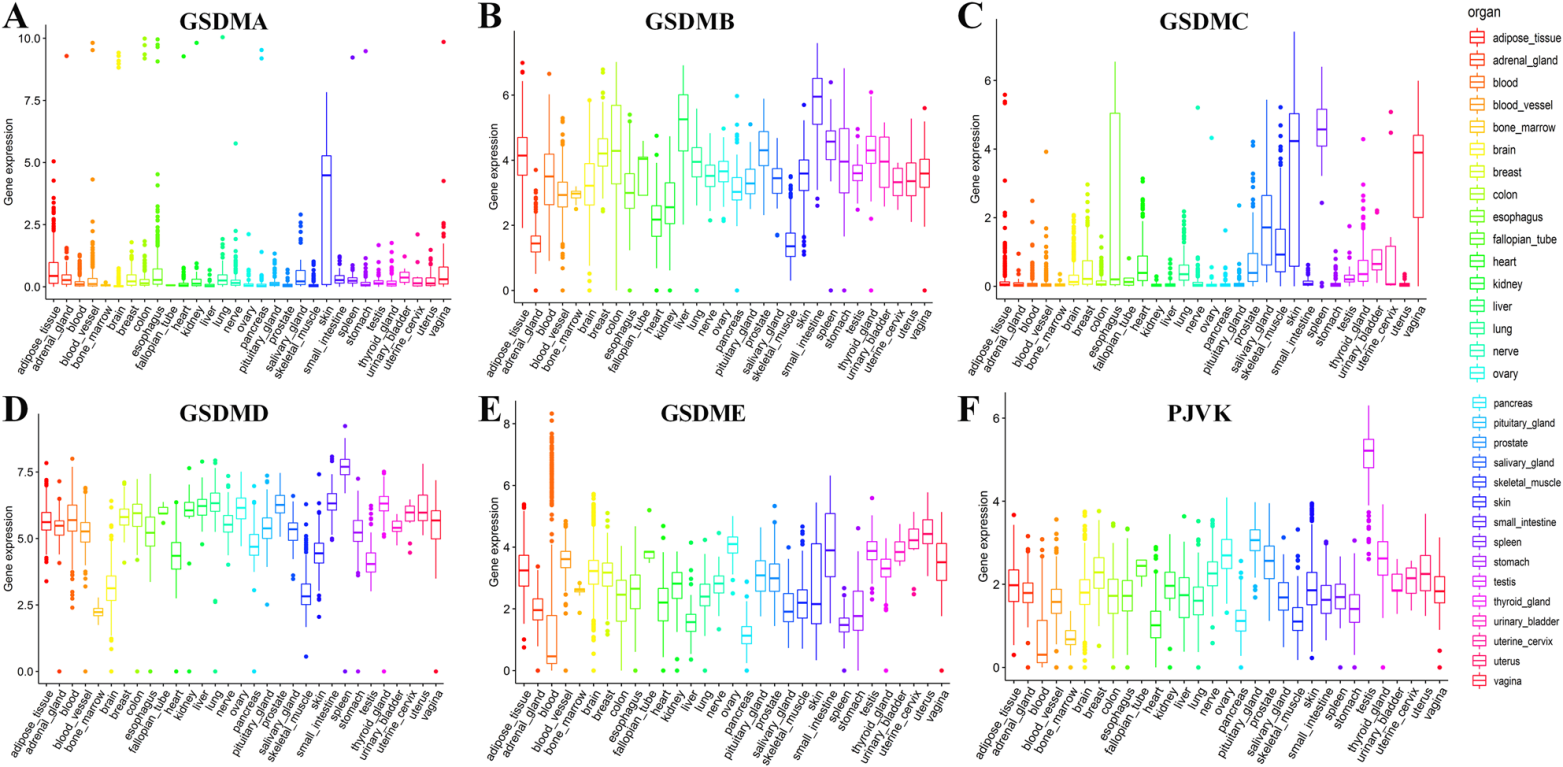
Expression levels of gasdermin family genes in normal tissues from the genotype-tissue expression (GTEx) database. GSDMA (**A**), GSDMB (**B**), GSDMC (**C**), GSDMD (**D**), GSDME (**E**), and PJVK (**F**). The Y axis represented the gene expression level, and the unit was log2 (TPM + 1).

The expression levels of gasdermin family genes were firstly investigated in different normal tissues. As illustrated in Fig. 1, GSDMA and GSDMC transcripts were faintly expressed in most tissues. Preferential enrichment of GSDMB and GSDMD was found in most tissues. However, there was a significant difference in the expression of GSDME and PJVK among different tissues. The testis had the most outstanding level of PJVK expression.

### Heterogeneous expression of gasdermin family genes across cancers

**Figure 2:**
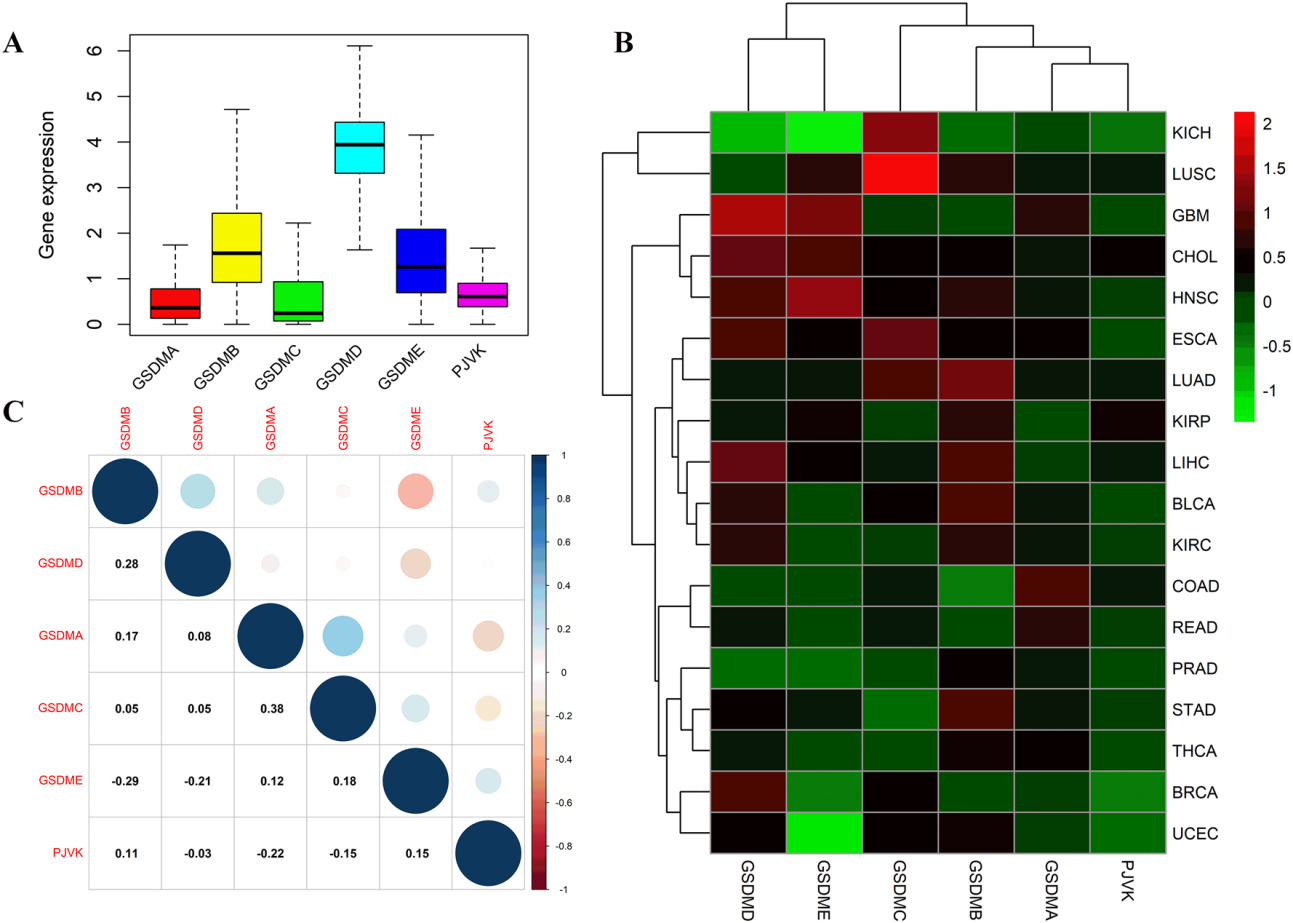
Gasdermin family gene expression levels and correlations across different cancer types from the TCGA database. The distinct expression of gasdermin family genes in various cancers (**A**). Heatmap depicting the expression differences of gasdermin family genes between tumor and surrounding normal tissues in different cancers. The red and green indicated the high or low expression, respectively. (**B**). The correlations among gasdermin family genes; Red dots represented negative correlations, whereas positive correlations were shown by blue dots (**C**).

In order to investigate the intrinsic gene expression patterns of gasdermin family genes, we further explored the gene expression in all 33 cancer types. As shown in Fig. 2A, our findings confirmed that the gene expression in each gasdermin family was significantly heterogeneous across cancers. Compared to the other gasdermin family genes, GSDMD had a higher average expression within all cancer types. GSDMA, GSDMC, and PJVK were expressed at low levels in all cancers. whereas, GSDMB and GSDME were moderately expressed in pan-cancer. As the main hallmark of tumorigenesis is the dysregulated gene expression in malignancies, our study further studied each gene expression of the gasdermin family individually across cancers. As shown in Fig. 2B, these genes showed varying amounts of expression in various malignancies. GSDMA and PJVK, for instance, were shown to be largely downregulated in various tumors. GSDMC was expressed at the highest levels in LUSC, GSDMD was expressed at the highest in GBM, and GSDME was expressed at the lowest in KICH and UCEC. Among these genes, GSDMA and GSDMC were the two genes with the most significant positive connection, whereas GSDMB and GSDME were the two genes with the most significant negative correlation (Fig. 2C).

### Comparative analysis of gasdermin family gene expression levels in tumor and normal tissues in pan-cancer

**Figure 3:**
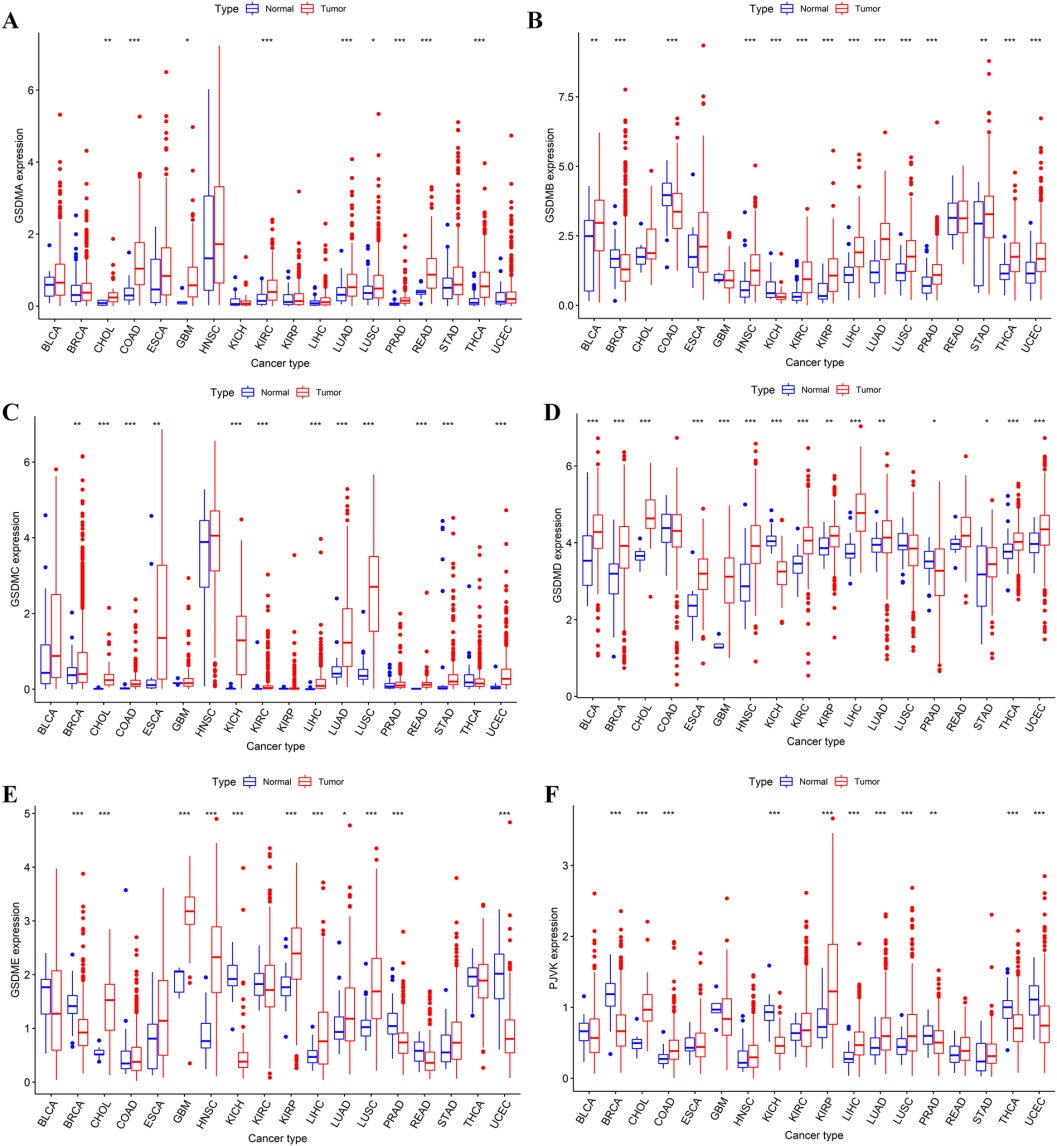
Comparisons of the gasdermin family gene expression levels between cancer and normal tissues from the TCGA database. The blue rectangle box represented genes expression levels in normal tissues, whereas the red rectangle box indicated gene expression levels in tumor tissues. *P* < 0.050, *P* < 0.010, and *P* < 0.001were denoted by “*”, “**”, and “***”, respectively. GSDMA (**A**), GSDMB (**B**), GSDMC (**C**), GSDMD (**D**), GSDME (**E**), and PJVK (**F**). The Y axis represented the gene expression level, and the unit was log2 (TPM + 1).

Subsequently, the richness of pan-cancer datasets from the TCGA was used to compare the expression levels of gasdermin family genes between tumor and surrounding normal tissue. Results showed that GSDMA was substantially upregulated in several cancer types, including CHOL, COAD, GBM, KIRC, LUAD, LUSC, PRAD, READ, and THCA (Fig. 3A). As displayed in Fig. 3B, GSDMB was lower expressed in BRCA, COAD, and KICH, while a higher GSDMB expression was found in BLCA, HNSC, KIRC, KIRP, LIHC, LUAD, LUSC, PRAD, STAD, THCA, and UCEC. Almost all cancer types showed substantial upregulation of GSDMC and GSDMD expression (Fig. 3C,D). The expression level of GSDME in tumor and adjacent normal tissue was significantly different (Fig. 3E). GSDME expression was decreased in BRCA, KICH, PRAD, and UCEC. In contrast, CHOL, GBM, HNSC, KIRP, LIHC, LUAD, and LUSC had a greater GSDME expression. Moreover, PJVK expression was significantly increased in CHOL, COAD, KIRP, LIHC, LUAD, and LUSC. PJVK expression downregulation was found in BRCA, KICH, PRAD, THCA, and UCEC (Fig. 3F).

**Figure 4:**
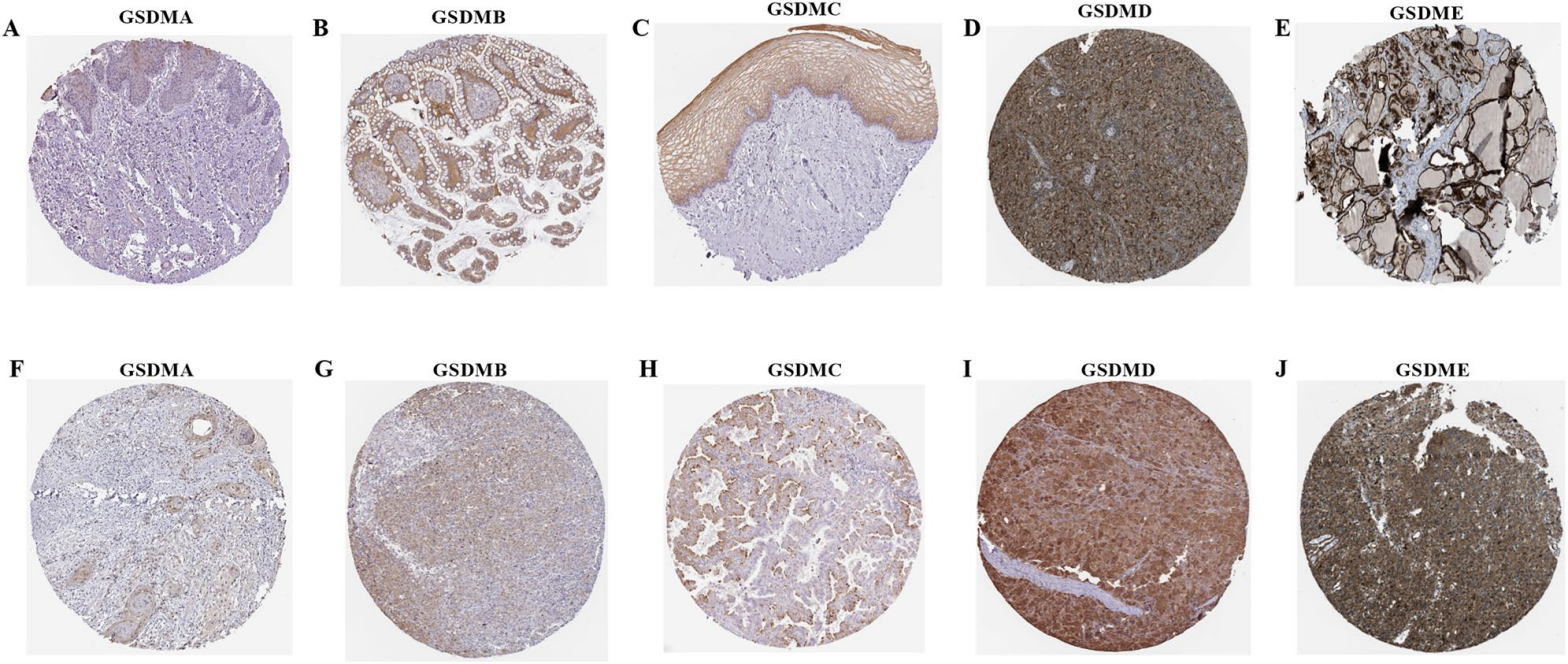
Representative IHC images of gasdermin family genes high expression in normal and tumor tissues. GSDMA expression in skin (**A**); GSDMB expression in small intestine (**B**); GSDMC expression in vagina (**C**); GSDMD expression in spleen (**D**); GSDME expression in thyroid gland (**E**); GSDMA expression in head and neck squamous cell carcinoma (**F**); GSDMB expression in stomach adenocarcinoma (**G**); GSDMC expression in lung squamous cell carcinoma (**H**); GSDMD expression in liver hepatocellular carcinoma (**I**); GSDME expression in glioma (**J**).

Subsequently, we analyzed the immunohistochemistry (IHC) results provided by the Human Protein Atlas (HPA) database. The IHC results showed the gasdermin family proteins level were similar with those genes expression results from TCGA database. Results showed gasdermin family genes were mainly distributed in cytoplasm and membrane. GSDMA was low expression in in most tissues except skin. High GSDMB and GSDMD expression were found in digestive tract tissues. Meanwhile, GSDMA, GSDMB, GSDMC, GSDMD, and GSDME were high expression in HNSC, STAD, LUSC, LIHC, and GBM, respectively. Representative IHC images were showed in Fig. 4.

### Prognostic significance of gasdermin family genes in pan-cancer

**Figure 5:**
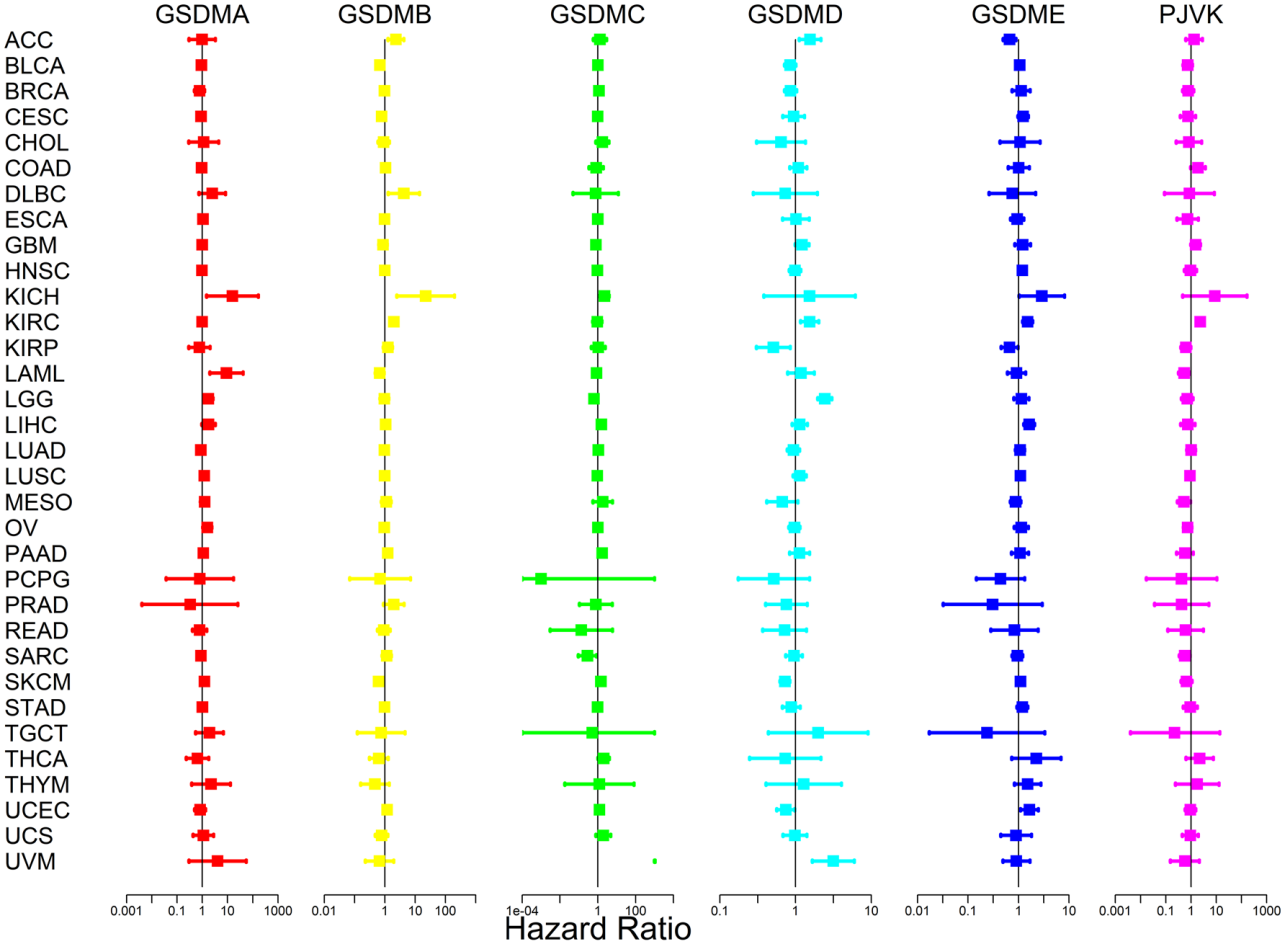
Univariate Cox proportional hazard regression showing correlations of gasdermin family gene expression with overall survival in pan-cancer; High risk was denoted by a hazard ratio > 1, whereas low risk was denoted by a hazard ratio < 1.

The prognostic risk of the gasdermin family genes in pan-cancer was then investigated using a univariate Cox proportional hazards regression model. Results revealed that aberrant expression of gasdermin family genes was closely associated with prognosis (Fig. 5, Table 1). GSDMA played a prognostic risk factor in KICH, LAML, LGG, OV, and SKCM. After *p*-value adjustment, the cancer types significantly associated with GSDMB were ACC, BLCA, DLBC, KICH, KIRC, LAML, PAAD, and SKCM. Besides, the upregulated expression of GSDMC was significantly related to poor prognosis in KICH, LIHC, PAAD, SKCM, THCA, and UVM, and good prognosis in LGG and SARC. Patients with KIRP, SKCM, and UCEC may have a better chance of surviving if GSDMD expression was increased. Conversely, high GSDMD expression was significantly related to poor prognosis in ACC, KIRC, LGG, and UVM patients. GSDME functioned as a high-risk gene in HNSC, KICH, KIRC, LIHC, and UCEC. Meanwhile, GSDME acted as a low-risk gene in ACC and KIRP. PJVK played a protective prognosis factor in LAML, SARC, MESO, and KIRP. In contrast, PJVK was a detrimental prognostic factor in KIRC. Consequently, our findings showed that the gasdermin family genes performed various roles in cancer progression and specific functions in distinct malignancies.

**Table 1:** Prognostic analysis of gasdermin family gene expression using the Cox method across different types of cancers.

Gene	Cancer	HR	HR.95L	HR.95H	*P*-value
GSDMA	KICH	15.578	1.474	164.631	0.022
	LAML	9.043	1.984	41.227	0.004
	LGG	1.738	1.137	2.655	0.011
	OV	1.581	1.057	2.366	0.026
	SKCM	1.220	1.057	1.408	0.006
GSDMB	ACC	2.315	1.262	4.245	0.007
	BLCA	0.682	0.595	0.781	< 0.001
	DLBC	4.213	1.285	13.809	0.018
	KICH	22.224	2.488	198.562	0.006
	KIRC	1.976	1.610	2.423	< 0.001
	LAML	0.672	0.483	0.936	0.019
	PAAD	1.240	1.037	1.482	0.018
	SKCM	0.618	0.455	0.839	0.002
GSDMC	KICH	2.251	1.271	3.989	0.005
	LGG	0.633	0.431	0.930	0.020
	LIHC	1.556	1.122	2.158	0.008
	PAAD	1.716	1.317	2.235	< 0.001
	SARC	0.283	0.094	0.849	0.024
	SKCM	1.469	1.184	1.822	0.000
	THCA	2.051	1.038	4.052	0.039
	UVM	1.673E + 08	1.068E + 03	2.621E + 13	0.002
GSDMD	ACC	1.553	1.118	2.159	0.009
	KIRC	1.538	1.165	2.032	0.002
	KIRP	0.510	0.305	0.853	0.010
	LGG	2.438	1.967	3.020	< 0.001
	SKCM	0.724	0.624	0.839	< 0.001
	UCEC	0.743	0.565	0.977	0.034
	UVM	3.150	1.661	5.974	0.000
GSDME	ACC	0.665	0.496	0.893	0.007
	HNSC	1.196	1.021	1.400	0.027
	KICH	2.898	1.032	8.138	0.043
	KIRC	1.517	1.210	1.901	0.000
	KIRP	0.663	0.452	0.973	0.036
	LIHC	1.630	1.277	2.081	< 0.001
	UCEC	1.645	1.098	2.464	0.016
PJVK	KIRC	2.317	1.623	3.306	< 0.001
	KIRP	0.626	0.395	0.992	0.046
	LAML	0.537	0.319	0.903	0.019
	MESO	0.525	0.289	0.954	0.034
	SARC	0.563	0.343	0.924	0.023

Given the dysregulated expression of gasdermin family genes in almost all cancer types, we further looked into the prognostic significance of gasdermin family genes in pan-cancer. We divided the patients into high- or low-expression groups based on the median of each gasdermin family gene expression level and then investigated the associations of gasdermin family gene expression with the prognosis in pan-cancer. As shown in Supplementary Fig. 1, KM survival curves displayed that gasdermin family genes were substantially associated with overall survival (OS) in various malignancies. Survival analysis showed that the low GSDMB expression was associated with a worse OS prognosis for BLCA, SKCM, and USC, whereas the high GSDMB expression was significantly related to a poorer prognosis of OS for KIRC (Supplementary Fig. 1A-D). OS survival analysis also revealed that GSDMC was a protective factor for patients with COAD and LGG, and a risk factor for BRCA, KICH, KIRC, PAAD and UVM (Supplementary Fig. 1E-K). Moreover, the result showed that GSDMD was a risk factor for patients with ACC, LGG, and UVM, and a protective factor for those with BLCA, SKCM, and UCEC (Supplementary Fig. 1L-Q). GSDME was proven to be risk against three kinds of cancer: KIRC, LIHC, and STAD. Contrarily, GSDME played a protective role in ACC (Supplementary Fig. 1R-U). OS analysis revealed that a higher PJVK expression was associated with a worse prognosis for KIRC and a better prognosis for LAML, MESO, PAAD, and SARC (Supplementary Fig. 1 V-Z).

### Genetic alteration analysis of gasdermin family genes in pan-cancer

**Figure 6:**
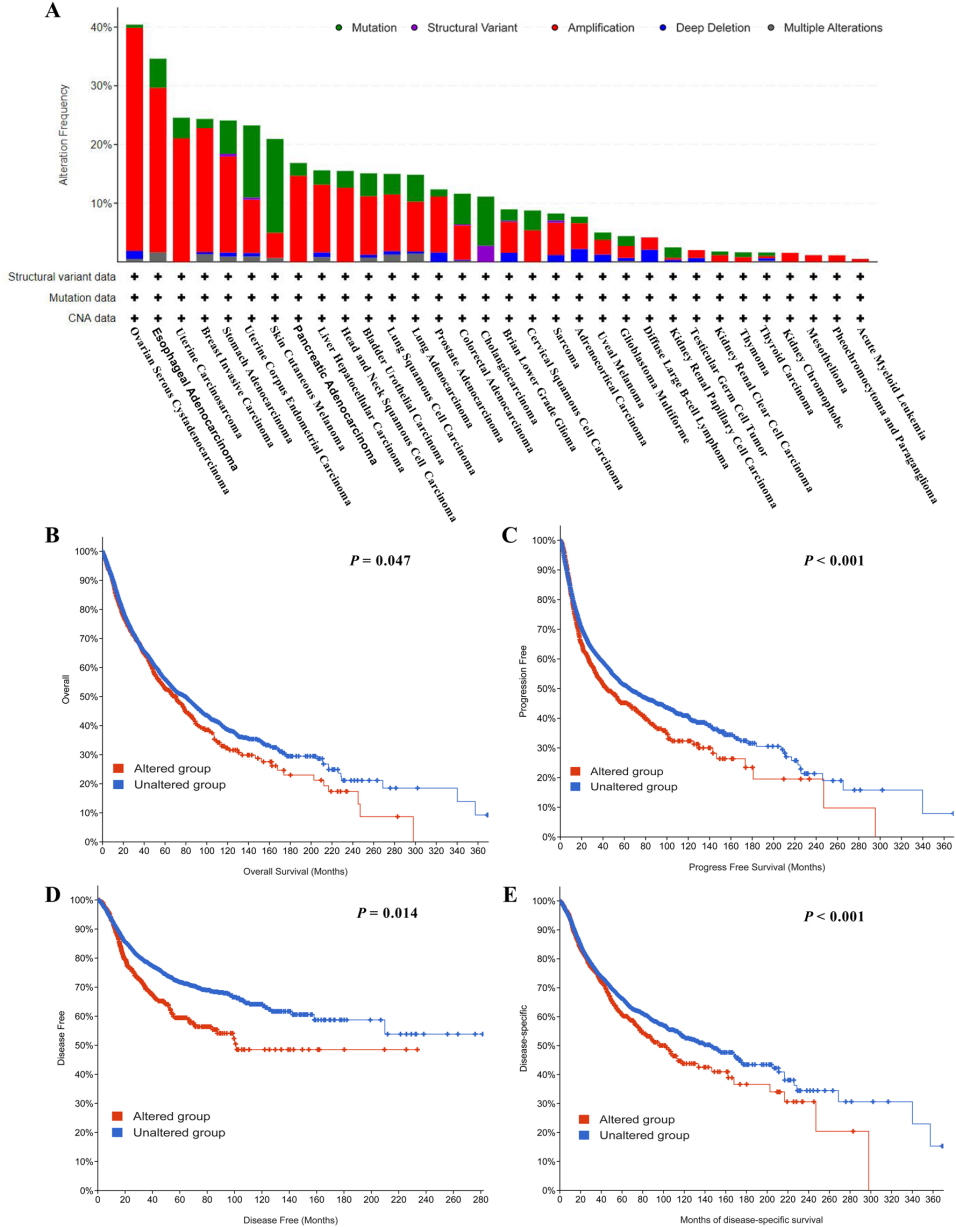
Genetic alteration analysis of the gasdermin family gene in pan-cancer. The genetic alteration type and frequency of the gasdermin family gene in pan-cancer (**A**). The potential correlations of the gene mutation status with overall survival (**B**), progression-free survival (**C**), disease-free survival (**D**), and disease-specific survival (**E**).

Using the Cbioportal for Cancer Genomics (http://www.cbioportal.org), we investigated the genetic alteration of gasdermin family genes in pan-cancer (Fig. 6A). The greatest prevalence of gasdermin family gene mutations was found in ovarian serous cystadenocarcinoma (40%), followed by esophageal adenocarcinoma (35%). In nearly all malignancies, “amplification” was the most common kind. Moreover, the associations of the gasdermin family gene alteration with relevant survival prognosis were investigated in pan-cancer. KM curves confirmed that the gasdermin family gene mutation group had shorter overall survival, progression-free survival, disease-free survival, and disease-specific survival than the non-mutation group (Fig. 6B–E).

### Associations of gasdermin family gene expression with tumor microenvironment and immune subtype in pan-cancer

**Figure 7:**
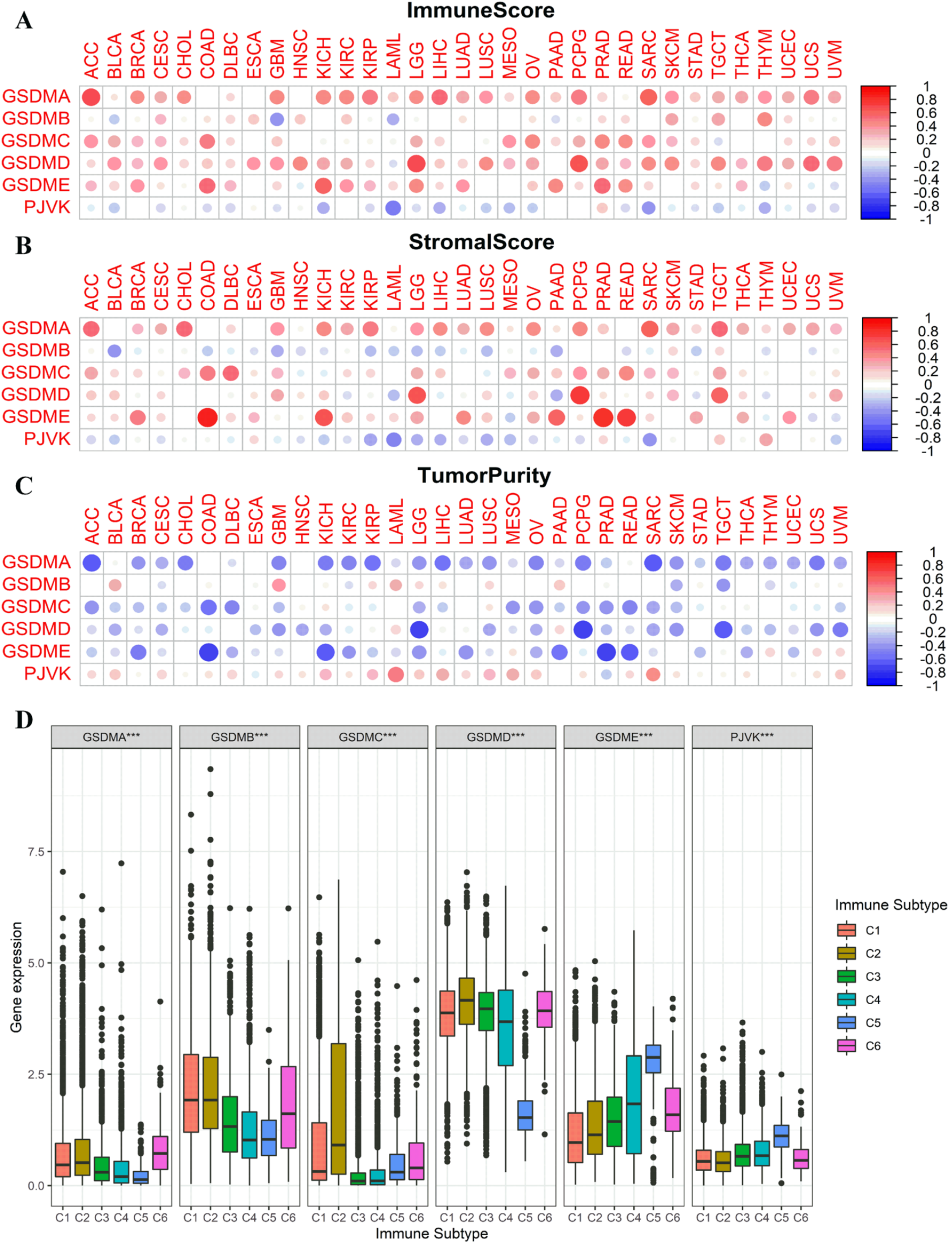
Associations of the gasdermin family gene expression with tumor microenvironment and immune infiltrate subtypes in pan-cancer. Correlation matrix plots showing the associations of gasdermin family gene expression with the immune score (**A**), stromal score (**B**), and tumor purity (**C**) of all the cancers; The size of the dots represented the absolute value of the correlation coefficients; The bigger the size is, the stronger the correlation would be; The red and blue represented the positive or negative correlation, respectively. Correlations of gasdermin family genes expression with immune infiltrate sub-types in all the malignancies (**D**); *P* < 0.050, *P* < 0.010, and *P* < 0.001were denoted by “*”, “**”, and “***”, respectively.

The tumor microenvironment has been widely recognized for crucial in tumorigenesis, malignant progression, metastasis, and multidrug resistance. Hence, we further investigated the associations of gasdermin family gene expression with tumor microenvironment across cancers. ESTIMATE algorithm was performed to quantify the immune cell infiltration (immune score), tumor purity, and stromal content (stromal score) in pan-cancer. The results showed gasdermin family gene expression was significantly correlated to immune score (Fig. 7A and Supplementary Table 1), stromal score (Fig. 7B and Supplementary Table 2), and tumor purity (Fig. 7C and Supplementary Table 3) across various cancer types, with the degree of the correlations varying greatly. Given the high associations between gasdermin family gene expression and tumor microenvironment, it was appropriate to look at gasdermin family gene expression with immune subtype in pan-cancer. Results showed that GSDMB was significantly associated with immune infiltration types C1, C2, and C6. GSDMA and GSDMC were relatively low expressed in all six types of immune infiltrate types. Except in C5, GSDMD presented strong expression in other immune infiltrate types. When compared to other types, GSDME and PJVK were higher expressed in C5 (Fig. 7D).

### Associations of gasdermin family gene expression with tumor mutational burden and microsatellite instability

**Figure 8:**
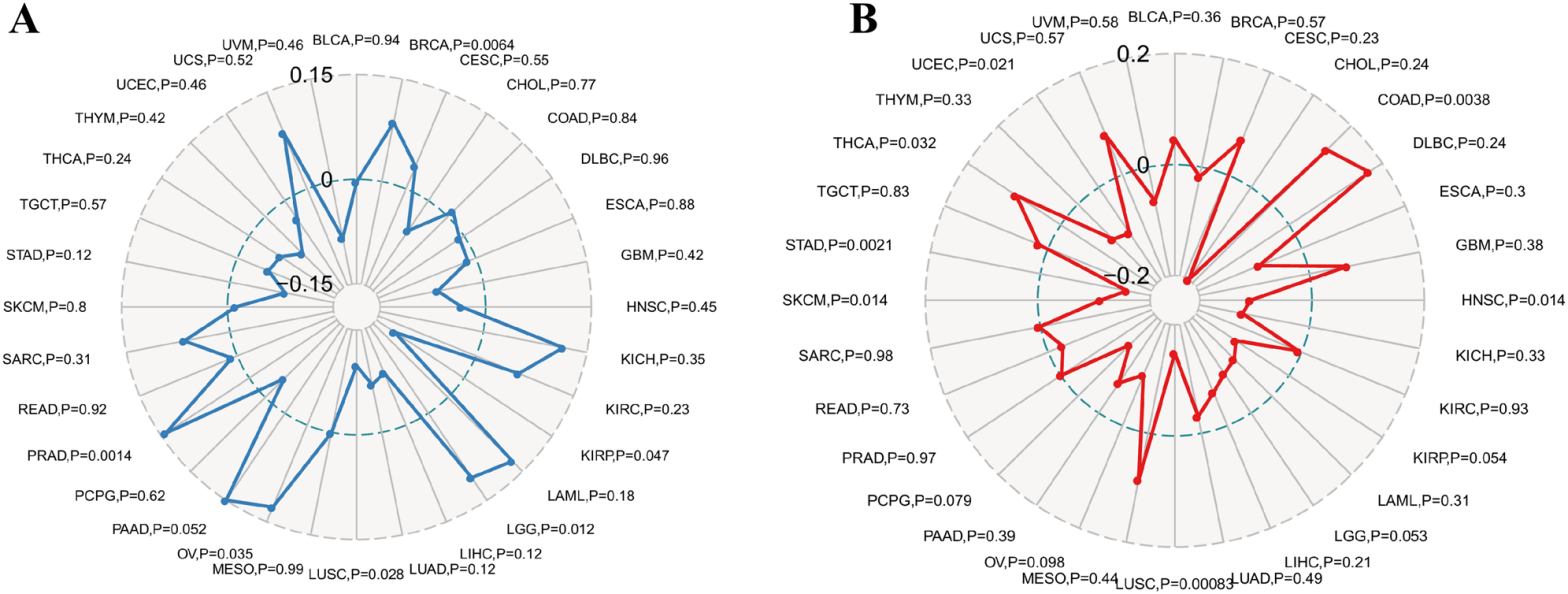
Correlation analysis of GSDMA expression with tumor mutational burden (TMB) (**A**) and microsatellite instability (MSI) (**B**) using Spearman’s rank correlation coefficient.

As shown in Fig. 8A and Supplementary Fig. 2A-E, correlation analysis showed that GSDMA was positively correlated with TMB in BRCA, LGG, OV, and PRAD and negatively correlated with KIRP and LUSC. Besides, GSDMB, GSDMC, GSDMD, and GSDME were significantly associated with TMB in more than ten types of tumors. Interestingly, PJVK was mainly negatively correlated with TMB in BRCA, CESC, ESCA, LUAD, PRAD, SARC, THCA, and UCEC.

When the relationships of gasdermin family gene expression with MSI were investigated, the correlations varied markedly among different cancer types (Fig. 8B and Supplementary Fig. 2 F-J). Higher expression of GSDMA was correlated with higher MSI in COAD and THCA and lower MSI in HNSC, LUSC, SKCM, STAD, and UCEC. Besides, higher GSDMB, GSDMD, and PJVK expressions were mainly associated with higher MSI in most tumors. GSDMC was positively correlated with MSI in BRCA, COAD, GBM, and LUSC and negatively correlated with CHOL, ESCA, and SKCM. At the same time, GSDME was negatively associated with MSI in GBM, STAD, and LUAD and positively correlated with COAD and SARC.

### Correlations of gasdermin family gene expression with immune checkpoints genes in pan-cancer

**Figure 9:**
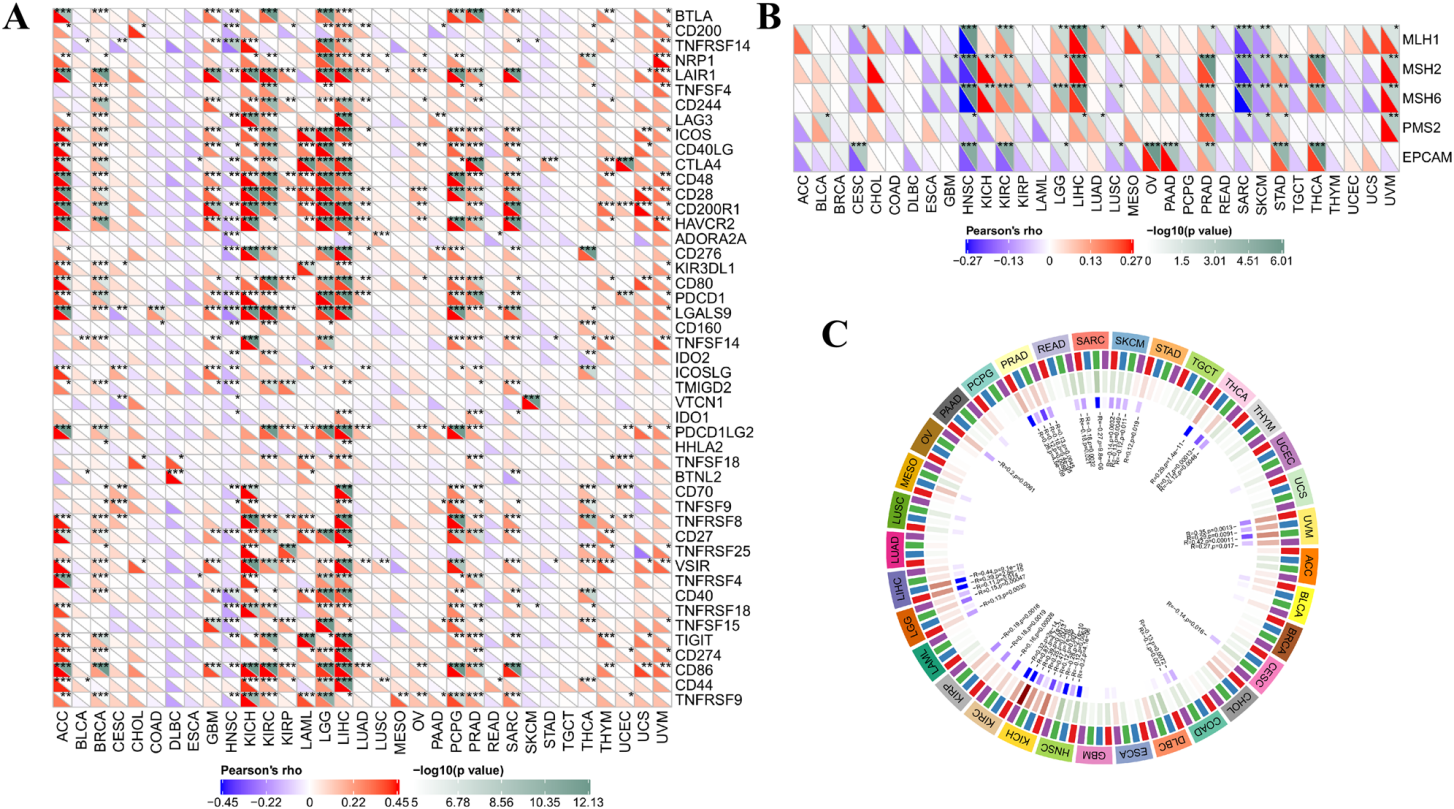
Correlation analysis of GSDMA expression with the expression levels of 40 common immune checkpoint genes (**A**), DNA mismatch repair (MMR) genes (**B**), and methyltransferases (**C**) in pan-cancer; The red and blue represented the positive or negative correlation, respectively (**A**, **B**); Red, blue, green, and purple represented DNMT1, DNMT2, DNMT3a, or DNMT3b, respectively (**C**). *P* < 0.050, *P* < 0.010, and *P* < 0.001were denoted by “*”, “**”, and “***”, respectively.

Given the significant correlations of gasdermin family gene expression with tumor microenvironment and immune subtype, we next explored the associations between gasdermin family gene expression and common immune checkpoints genes. As shown in Fig. 9A and Supplementary Fig. 3, the expression levels of gasdermin family genes were significantly correlated with the expression levels of immune checkpoints genes in various types of tumors. Interestingly, results confirmed that GSDMA was positively correlated with more than 30 immune checkpoint biomarkers in KICH, KIRC, LGG, and LIHC. Moreover, the GSDMD expression level was positively correlated with the expression levels of LGALS9 in all types of tumors. These results suggested that abnormal expression of the gasdermin family gene played a vital role in mediating the tumor immunity pattern.

### Correlation analysis of gasdermin family gene expression with the expression of DNA mismatch repair gene and methyltransferase in pan-cancer

The function loss of MMR genes might result in higher somatic mutations and tumorigenesis. We further investigate the detailed correlations of gasdermin family gene expression with MMR gene mutation levels to evaluate the effect of the gasdermin family gene on tumorigenesis. Results confirmed that the correlations varied markedly across different types of cancers (Fig. 9B and Supplementary Fig. 4A-E). GSDMA was negatively correlated with all the MMR gene expression levels in HNSC and positively associated with PRAD gene expressions. In contrast, the expressions of GSDMB, GSDMC, and GSDMD were significantly correlated with the MMR gene expression only in some specific tumors. Almost all the MMR gene expressions were positively associated with GSDME and PJVK expression levels except for a few tumors.

The DNA methylation status alteration is a significant factor of tumorigenesis. Hence, we investigated the correlations between gasdermin family gene expression and 4 DNA methyltransferases in pan-cancer. Figure 9C and Supplementary Fig. 4F-J showed that the expressions of gasdermin family genes were closely associated with one or more methyltransferases in specific tumors, among which GSDME and PJVK were particularly prominent.

### Associations of gasdermin family gene with tumor stemness and drug sensitivity

**Figure 10:**
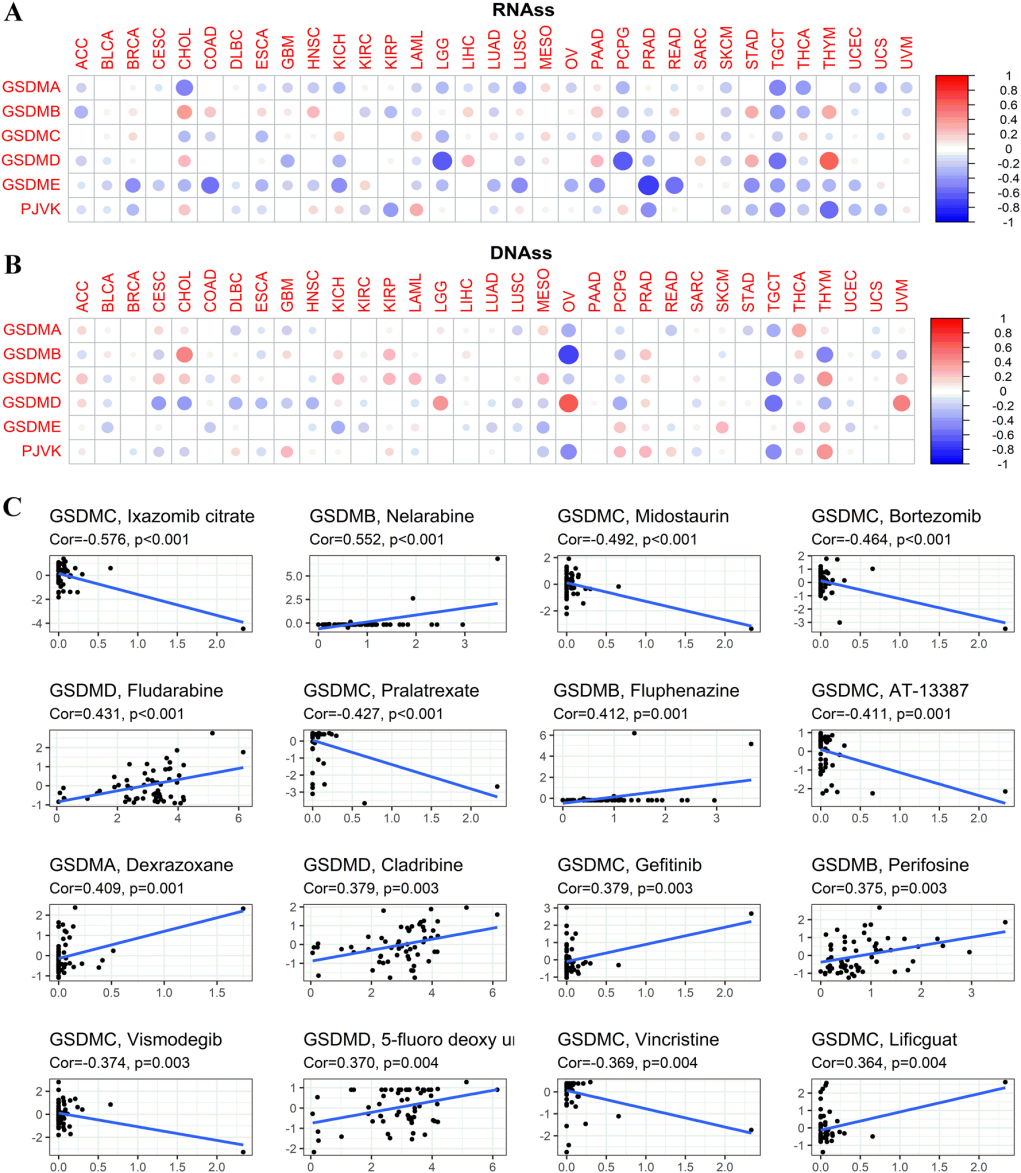
Correlations of gasdermin family gene expression with tumor stemness and treatment susceptibility. The associations between gasdermin family gene expression with tumor stemness scores, including RNAss (**A**) and DNAss (**B**); The size of the dots represented the absolute value of the correlation coefficients; The bigger the size is, the stronger the correlation would be; The red and blue represented the positive or negative correlation, respectively. Spearman’s correlation showing the associations between gasdermin family gene expression and drug sensitivity using NCI-60 cell line data (**C**).

As the tumor progresses, tumor cells would lose their phenotypes and gain progenitor and stem cell-like features to a certain extent. Tumor stemness score is mainly evaluated from RNA stemness score (RNAss) and DNA stemness score (DNAss) viewpoints. Gasdermin family gene was proven to be positively or negatively correlated to RNAss and DNAss to different degrees among cancer types (Fig. 10A,B).

We further investigated the potential associations between gasdermin family gene expression and drug sensitivity in 60 human cancer cell lines (NCI-60) from the CellMiner™ database after finding substantial correlations of gasdermin family genes with tumor stemness scores. Results confirmed that the upregulated expression of GSDMC was significantly related to reduced chemotherapeutic drugs sensitivity in distinct cell lines. Contrarily, the decreased expressions of most of the remaining gasdermin family genes were significantly related to the increased sensitivity of distinct cell lines to chemotherapeutic drugs (Fig. 10C).

### Associations of gasdermin family gene expression with pathologic grade, immune subtype, stemness score, and the tumor microenvironment in selected types of cancer

**Figure 11:**
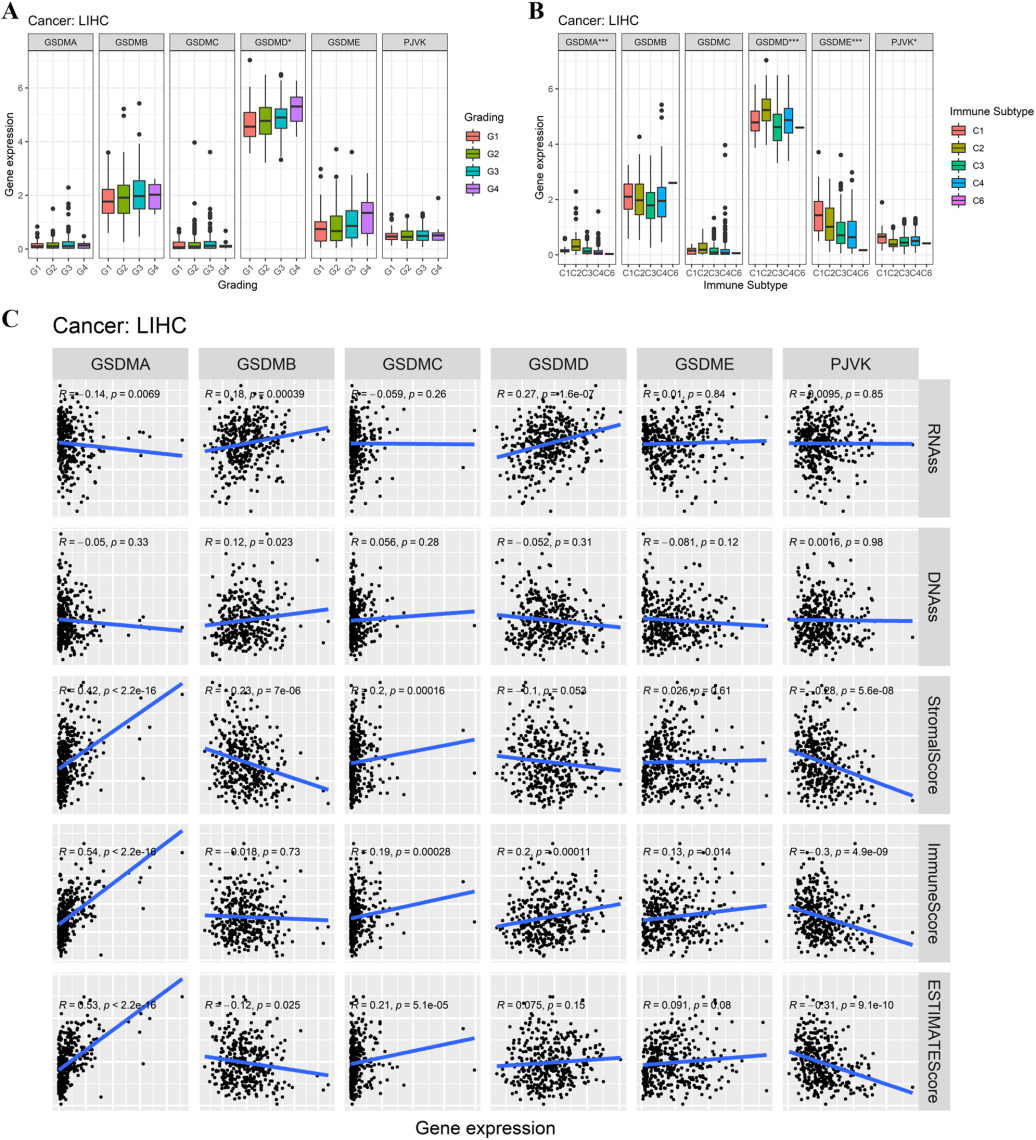
Associations of gasdermin family gene expression with the clinical grade, immune subtype, stemness score, and tumor microenvironment in LIHC. Gasdermin family genes expression levels in various clinical grades (**A**). Associations of gasdermin family gene expression with distinct immune infiltrate subtypes (**B**). Linear regression analysis showing the correlations of gasdermin family gene expression with stemness score and the tumor microenvironment (**C**). *P* < 0.050, *P* < 0.010, and *P* < 0.001were denoted by “*”, “**”, and “***”, respectively.

To back up our findings, we conducted comprehensive and systematic investigations of the gasdermin family gene in LIHC and PAAD. We firstly investigated whether gasdermin family gene expression differed with various clinical grades in LICH and PAAD. Results showed that GSDMD expression levels increased with tumor grade in LICH (Fig. 11A). Besides, as PAAD grade advanced, the expression levels of GDSMB, GDSMC, GDSMD, and GDSME consistently rose as well (Supplementary Fig. 5A). When gasdermin family gene expression associated with immune subtype was investigated in LICH, results showed that gasdermin family genes except GSDMB and GSDMC were significantly related to the immune subtype (Fig. 11B). The expression levels of GSDMB, GSDMC, and GSDMD were associated with different kinds of immune infiltrates in PAAD (Supplementary Fig. 5B).

Correlation analysis indicated that GSDMB and GSDMD expressions were positively associated with RNAss, whereas the expression of GSDMA was negatively related to RNAss in LIHC. Furthermore, GSDMB expression was positively correlated to DNAss in LIHC (Fig. 11C). In LIHC, correlation analysis of gasdermin family gene expression with tumor microenvironment revealed that GSDMA and GSDMC expressions were positively related to stromal score, whereas GSDMB and PJVK expressions were negatively associated with the stromal score. Additionally, the expressions of GSDMA, GSDMC, GSDMD, and GSDME were positively correlated with immune score and ESTIMATE score, whereas PJVK expression had a negative correlation with immune score and ESTIMATE score in LIHC. When the correlation analysis of gasdermin family gene expression with tumor microenvironment was performed in PAAD, we found that all members except for GSDMC showed a significant association with RNAss (Supplementary Fig. 5C). No significant correlation was found between gasdermin family gene expression and DNAss in PAAD. Moreover, the stromal score, immune score, and ESTIMATE score were negatively associated with GSDMB and GSDMD and positively related to GSDME. Besides, the PJVK expression was also negatively associated with the stromal score in PAAD.

### Gene sets enrichment analysis

**Figure 12:**
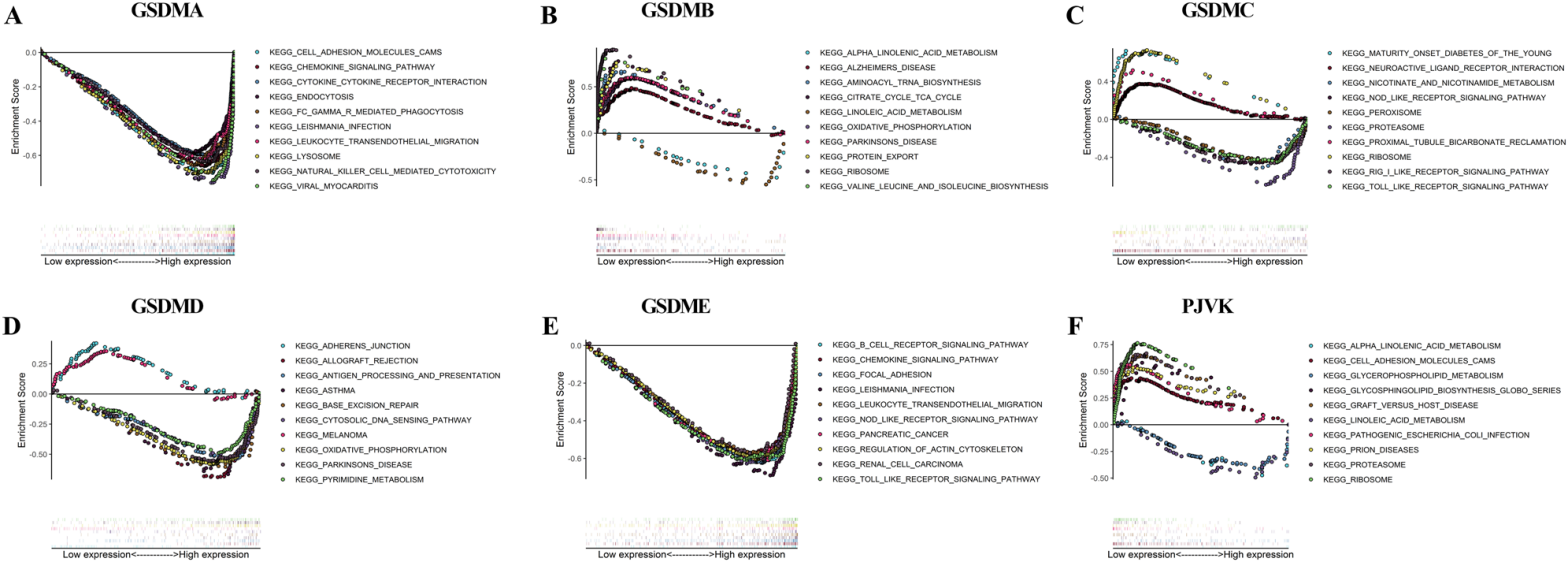
Gene set enrichment analysis (GSEA) of gasdermin family gene associated with signaling pathways in KEGG dataset. GSDMA (**A**), GSDMB (**B**), GSDMC (**C**), GSDMD (**D**), GSDME (**E**), and PJVK (**F**). Each line representing one particular signaling pathway with unique color, and low-expressed genes lay on the left approaching the origin of the coordinates, and the high-expressed located in the right of X-axis.

In order to investigate the function of gasdermin family gene in cancers, we divided all the human pan-cancer samples into high- and low-expression groups based on the median value and analyzed the significant enrichment pathways in KEGG and hallmark datasets by GSEA. As shown in Fig. 12 and Supplementary Fig. 6, the top 10 signaling pathways significantly enriched in both databases have been listed. Figure 12A and Supplementary Fig. 6Ashowed that the significant signaling pathways were enriched in the group with high GSDMA expression and mainly associated with immune or inflammatory related cytokines and cells activities. Besides, the differential expressions of GSDMB and GSDMC were mainly related to metabolic-related pathways and disease (Fig. 12B-C and Supplementary Fig. 6B-C). The differential expression of GSDMD was discovered to be involved in drug response, DNA and base excision repair, metabolism, allograft rejection, and cytokine activity (Fig. 12D and Supplementary Fig. 6D). Figure 12E and Supplementary Fig. 6 E verified that high GSDME expression was mainly associated with regulation of signaling pathways involved in tumor immune microenvironment and carcinogenesis-related pathways. As shown in Fig. 12F and Supplementary Fig. 6F, PJVK was also confirmed to participate in tumor immune microenvironment remodeling.

## Discussion

The pan-cancer analysis could reveal commonness and differences among tumors, providing new insight into cancer prevention and therapeutic drug development. Recently, increasing studies have focused on the pan-analysis of the whole genome to investigate the associations of abnormal gene expression with tumorigenesis, cancer progression, and prognosis, which might play significant roles in the early diagnosis and treatment of cancer^23–25^. Gasdermin family gene was initially found to be mainly expressed in the upper digestive tract and skin^26^. According to the conserved N-terminal and C-terminal regions, six homologous members of the gasdermin family have been found in humans, namely GSDMA, GSDMB, GSDMC, GSDMD, GSDME, and PJVK^27^. Recently, pyroptosis is renamed as gasdermin-mediated programmed necrosis since the gasdermin family of pore-forming proteins play a key role in the process^28^. Pyroptosis, characterized by rapid membrane rupture, cell swelling with large bubbles and release of cellular proinflammatory contents, plays vital pathophysiological roles in atherosclerosis, diabetics, pathogen infections, and organ failures^29^. Moreover, gasdermin family genes express in stage-specific and tissue-specific manners^30^, indicating that they are involved in a series of physiological and pathological processes including epithelial cell development, apoptosis, and immune-related disorders^17,18,31–33^. GSDME was even identified as the substrate of caspase-3 that executes pyroptotic cancer cell death and also induces the systemic side effects of chemotherapy drugs^21^. Besides, the cleavage of gasdermin triggers the inflammatory tumour microenvironment and enhances the efficacy of cancer immunotherapy^34^. Hence, gasdermin family genes might have huge potential to exploit for biomedical applications in cancer therapy. . Although several studies have investigated the relationships between the gasdermin family gene and cancers^13,35^, the roles of the gasdermin family gene in cancer pathogenesis and underlying mechanisms deserve to be investigated in pan-cancer. Hence, we performed the first comprehensive pan-cancer analysis of the gasdermin family gene.

We first used the GTEx database to investigate the expression levels of the gasdermin family gene in normal tissue. Significant differences were found in the gasdermin family gene expression among different tissues, indicating that the gasdermin family genes perform specific functions in various organizations. The expression levels of gasdermin family genes were further compared between tumor and adjacent normal tissues in pan-cancer using the TCGA database. Results confirmed that GSDMA was substantially overexpressed in eight cancers tissues compared to normal tissues, whereas GSDMB and PJVK had a high inter-tumor heterogeneity between paracancerous and tumor tissues. GSDMC and GSDMD were found to be significantly upregulated in almost all cancer types. GSDME was shown to be underexpressed in four cancer types and overexpressed in seven others. Besides, the gasdermin family genes were expressed differently in pan-cancers. Therefore, our study shed insights into the roles of the gasdermin family genes as biological indicators of the tumor with prognostic significance, and it might contribute to advance targeted treatment research for the gasdermin family gene.

Given that the dysregulated expression of gasdermin family genes in almost all cancers, we further examined the relationship between gasdermin family gene expression and the prognosis in pan-cancer. Survival analysis showed that the gasdermin family genes played various roles in cancer progression and might provide specific functions in different cancers. Moreover, these genes function as a protective or risk factor in different tumors. Previous studies showed that overexpression of GSDMB promoted aggressiveness of breast cancer with poor prognosis, and inhibiting GSDMB expression enhanced the cancer sensitivity to trastuzumab and limited cancer cell invasion in vitro^36–38^. Hence, GSDMB was considered as an oncogene. According to our findings, overexpression of GSDMB was also associated with poor prognosis in several cancers. Saekiet al. reported that GSDMC overexpression inhibited the esophageal squamous cell carcinomas cells growing^30^. Contrarily, Miguchi et al. confirmed that GSDMC overexpression enhanced cell proliferation and xenograft tumor growth^39^. Whether the GSDMB is an oncogene or not would be difficult to answer in the future. Consistent with the difficulty, our study revealed that GSDMC might function as a risk or protective factor in different malignancies. When we further investigated the associations of family members with prognosis, we discovered that cancer type had a risk or protective impact. The genetic alterations of the gasdermin family gene were then investigated in pan-cancer. The primary mutation type was "amplification". The mutation frequencies seemed to be very high, particularly in ovarian serous cystadenocarcinoma and esophageal adenocarcinoma. Moreover, KM curves confirmed that the mutation group had a poorer prognosis than the non-mutation group. Therefore, our findings confirmed that the gasdermin family genes might be used as a pan-cancer predictive biomarker.

Currently, immunotherapy is being researched as a tumor treatment that restarts and maintains the tumor-infiltrating immune cells cycle to restore normal anti-tumor immune activity^40^. Although multimodal therapies, including adoptive T cell therapy and immune checkpoint inhibitors, have significantly improved the prognosis, the survival benefit remains limited. One of the main reasons is that cancer cells might successfully evade the body’s immune system caused by the inherently immunosuppressive microenvironment^41–43^. Pyroptosis causes cell enlargement, plasma membrane rupture, chromatin fragmentation, and the release of intracellular proinflammatory components such as inflammatory vesicles, gasdermin proteins, and proinflammatory cytokines in the presence of unfavorable conditions^44^. Increasing evidence indicated that the multiple signaling pathways and inflammatory contents were closely associated with tumorigenesis^17,18^. Novel potential immunotherapy for pyroptosis has also been studied by a growing number of researchers^21,22^. in the study, we discovered that various degrees of the immune and stromal cell infiltrate correlated to gasdermin family gene expression in pan-cancer, with the degree of the association showing significant diversity. Besides, we also found that all members of the gasdermin family were closely related to different kinds of immune subtypes. Previous research showed that cancer patients with type C4 and C6 had a worse survival rate, while patients with type C3 and C5 had a better survival rate owing to favorable immune composition^45^. GSDMB was shown to be associated with C1, C2, and C6 subtypes, suggesting that it is more likely to be an oncogene. In contrast, GSDME and PJVK were more expressed in C5 than other types, suggesting that these genes mainly functioned as anti-oncogenes. These results indicated that individuals with diverse gasdermin family gene expressions might have variable clinical characteristics and immunotherapy responses due to immune and stromal cell composition variations. TMB, a latent biomarker to predict the response to immune checkpoint blockade, determined the immune-related prognosis of cancer^46,47^. Previous studies confirmed that MSI was correlated with clinicopathological features of high risk for cancer, including more tumor-infiltrating lymphocytes and increased TMB^48^. Indeed, correlation analysis showed that gasdermin family gene expressions were significantly associated with TMB and MSI in various cancers. Based on the above findings, we further investigate the associations of gasdermin family gene expression with more than 40 common immune checkpoint genes expressions. Results showed that the expression levels of gasdermin family genes were significantly correlated with the expression levels of immune checkpoints genes in various types of tumors. Therefore, gasdermin family genes might be potential biomarkers and play an indispensable role in tumor immunology.

What gasdermin family genes were associated with MMR genes mutation and DNA methyltransferase expression confirmed that abnormal gasdermin family gene expression could mediate tumorigenesis and progression by regulating the expression of MMR genes and methyltransferase. Tumor stemness was proven to be associated with tumor pathology, invasion, immune microenvironment content, and immunotherapy^49,50^. Besides, the tumor stemness score might help explain some of the tumor's intrinsic heterogeneity and even serve as a prognostic indicator^51^. We also found that the expression of gasdermin family genes was negatively or positively correlated with tumor stemness score. Hence, the gasdermin family gene might assist us in identifying individuals benefiting from stem cell-based immunotherapy effectiveness. Subsequently, correlation analysis revealed that upregulated GSDMC expression was significantly related to reduced chemotherapeutic drug sensitivity of distinct cell lines. Contrarily, the upregulated expressions of most of the remaining gasdermin family genes were significantly related to greater susceptibility to chemotherapeutic treatments. These findings shed new insights on the understanding of cancer immunotherapy and chemotherapy and indicated that gasdermin family genes might be used as potential targets for cancer treatment.

In order to validate our findings, we performed comprehensive and systematic investigations of gasdermin family genes in LIHC and PAAD. First, we found that gasdermin family gene expression levels increased with tumor grade advanced and were significantly correlated with immune subtypes. Consistent with the pan-cancer findings, gasdermin family genes were positively or negatively associated with tumor stemness indexes and tumor microenvironment. Subsequently, GSEA was performed to investigate the special functions and mechanism of the gasdermin family gene in pan-cancer. Results indicated the gasdermin family genes were widely associated with the regulation of signaling pathways mainly involved in tumor metabolism and immune microenvironment remodeling. The results confirmed that these genes influenced tumor biology, and microenvironment, and might be as the therapeutic targets in pan-cancer. The detection of gasdermin family genes had particular guiding significance for the clinical treatment.

Our research revealed the complicated roles of gasdermin family genes aberrant expression in tumorigenesis, progression, prognosis, and treatment in pan-cancer. Besides, we preliminarily explore the underlying mechanism and biological functions of the gasdermin family genes in various cancers. However, rigorous mechanistic interpretation from in vitro and vivo experiments might allow us to draw more general and accurate conclusions.

## Conclusion

Our study investigated the expression of gasdermin family genes in a broader view. It revealed the detailed associations with tumorigenesis, tumor progression, prognosis, tumor microenvironment, immune response, TMB, MSI, immune checkpoint genes, MMR genes, DNA methyltransferase, tumor stemness index, and specific therapeutic sensitivity in various malignancies. These findings may provide insights for further investigation of the gasdermin family genes as potential targets in pan-cancer. Our findings shed new insights into the understanding of immunotherapy and chemotherapy to cancer and confirmed that gasdermin family genes are potential therapeutic cancer targets in pan-cancer.

## Materials and methods

### Analysis of gene expression data

The expressions data of the gasdermin family genes in different normal tissues from healthy individuals were obtained from the genotype-tissue expression (GTEx) dataset on September 11, 2020 (Version 8). Gene expression RNA-seq data, clinical data, survival data, stemness scores based on mRNA expression and DNA methylation, and immunological subtype of 33 pan-caners were obtained from the TCGA database on March 31, 2021 (Version 29.0). The genotype-tissue expression (GTEx) and the Cancer Genome Atlas (TCGA) were extracted from the University of California Santa Cruz (UCSC) Xena browser (https://xenabrowser.net/datapages/). The difference in gasdermin family genes expressions between normal and tumor tissues was further investigated by combining the data for normal tissues from the GTEx database with the data from the TCGA database. Fragments per kilobase million (FPKM) values were transformed into transcripts per kilobase million (TPM) values, and were further log transformed for better comparisons between samples. As only 18 of 33 types tumors had normal tissue, we only compared the expression difference in the 18 types of tumors. The expression level and differential analysis were visualized using the "ggpubr" R package, and the Wilcoxon test determined the difference. The “pheatmap” R package drew heatmap to visualize the different expression levels of genes in pan-caner. Correlation analysis among the gasdermin family genes was conducted using the "corrplot" R package.

### Gasdermin family genes expression profiles analysis

According to integration of various omics technologies, HPA (http://www.proteinatlas.org/) is a program for mapping human proteins in cells, tissues and organs. To evaluate differences in gasdermin family genes’ expression at the protein level, IHC images of gasdermin family genes’ protein expression in normal tissues tumors tissues were downloaded from the HPA and analyzed.

### Survival analysis

All patients were respectively divided into low- and high-risk groups based on the median expression level of each member of the gasdermin family gene. The Kaplan–Meier (KM) method was used to examine the prognostic value across various malignancies, and the log-rank test was employed to assess the significance. Moreover, these family gene expression levels were treated as continuous variables to explore the prognostic value by COX analysis in pan-cancer. The KM method and COX analysis were performed and visualized by the "survival" and "survminer" R packages, and the forest plot was delineated by the “survival” and “forestplot” R packages.

### Genetic alteration analysis

The genetic alterations of the gasdermin family gene in pan-cancer were investigated from the “TCGA Pan-Cancer Atlas Studies” module of the cbioportal for Cancer Genomics (http://www.cbioportal.org). The genetic alteration concludes mutation, structural variant, amplification, deep deletion, and multiple alterations. The detailed information was displayed in the “Cancer Types Summary” section. KM curves were used to evaluate the associations of genetic alteration with prognosis in pan-cancer.

### Correlation analysis of gasdermin family gene expression with tumor microenvironment and immune subtype

ESTIMATE^52^ was performed to calculate the immune cell infiltration level (immune score), tumor purity, and stromal content (stromal score) in pan-cancer tissues. Then, spearman's method was performed to investigate the correlations between the gasdermin family gene expression and these scores across various cancers. Six immune subtypes were defined to measure immunological infiltrates in the tumor microenvironment^53^. The immune subtype consists of C1 (wound healing), C2 (IFN-g dominant), C3 (inflammatory), C4 (lymphocyte depleted), C5 (immunologically quiet), and C6 (TGF-b dominant). Variance analysis was further performed to investigate the relationship between the family gene expression and immunological infiltrate.

### Correlation analysis of gasdermin family gene with tumor mutational burden and microsatellite instability

TMB, the mutation density of tumor genes, is the number of mutations per million bases in tumor tissue, including base substitution, gene insertion, and gene coding and deletion errors. It is also defined as the average number of mutations in the tumor genome. Microsatellite instability (MSI) refers to the appearance of new microsatellite alleles in tumor tissue due to any change in the length of a microsatellite resulting from insertion or deletion of duplicate units compared with normal tissue. Spearman's rank correlation coefficient was used to explore the correlations of gene expression with TMB and MSI. Visualization analysis was performed by the "fmsb" R package.

### Correlation analysis of gasdermin family gene with immune checkpoint genes in pan-cancer

Currently, immunotherapy is a hot field of investigation for cancer treatment. Immune checkpoint inhibitors, as novel tumor immunotherapy agents, function as essential roles in tumor immunotherapy. The expression levels of common more than 40 immune checkpoint genes were obtained from the TCGA database, and the associations between gasdermin family gene expression and these immune checkpoint biomarkers were investigated based on spearman’s correlation analysis. What is more, neoantigen was encoded by a mutated gene from tumor cells. The mutation mainly concludes point mutation, deletion mutation, and gene fusion. Predicted neoantigen was calculated based on binding affinity, variant allele frequency, and antigenicity index values. Then, the number of neoantigens per tumor sample was respectively counted. Spearman’s correlation analysis further explored the associations of gasdermin family gene expression with the number of neoantigens.

### Association analysis of gasdermin family gene with mismatch repair gene mutation, DNA methyltransferase, and stemness score

MMR is an intracellular mismatch repair mechanism. The functions loss of the mismatch repair genes might lead to DNA replication errors that cannot be repaired, resulting in higher levels of somatic mutations and tumorigenesis. DNA methylation, a form of DNA chemical modification, could alter gene expression without changing the DNA sequence. DNA methylation could control gene expression by altering chromatin structure, DNA conformation, DNA stability, or the interaction between DNA and protein. Pearson correlation analysis was performed to estimate the correlations of the gasdermin family gene with the expression levels of five MMR genes (MLH1, MSH2, MSH6, PMS2, and EPCAM) and four methyltransferases (DNMT1, DNMT2, DNMT3A, and DNMT3B).

Subsequently, the stem cell-like properties of tumor cells were detected based on their transcriptome and epigenetic characteristics^49^. The stemness score concludes RNA stemness score (RNAss) and DNA stemness score (DNAss). RNAss was calculated based on mRNAs expression, whereas DNAss was measured based on the DNA methylation pattern. Spearman’s correlation analysis was utilized to assess the associations between the gasdermin family gene and cancer stemness scores.

### Drug sensitivity analysis of gasdermin family gene

Drug sensitivity analysis data was downloaded from the CellMiner™ interface (version:2021.2 (database:2.7)) (https://discover.nci.nih.gov/cellminer/). The database contains 60 human cancer cell lines (NCI-60) from nine types of tumors^54^. 262 FDA-approved or clinical trial medications were used in the drug sensitivity study. Linear regression analysis was conducted to investigate the drug sensitivity.

### Gene set enrichment analysis of gasdermin family gene in pan-cancer

GSEA was performed to investigate the special functions and mechanisms of the gasdermin family in pan-cancer. The method derives its function by analyzing gene sets to determine whether the gene set shows a statistically significantly difference between the high- and low-expression groups. Within the “Molecular Signatures Database” of Kyoto Encyclopedia of Genes and Genomes (KEGG) and hallmark gene sets by GSEA, underlying mechanisms and functions were studied. Using |Net enrichment score (NES)|> 1, *P* value < 0.050, and FDR *q* < 0.25 as the threshold, pathways were considered to be enrichment significant. The top ten terms of KEGG and Hallmark analyses were exhibited.

### Statistical analysis

The “Wilcox. test” method was utilized to compare differences between two groups, and one-way analysis of variance (ANOVA) was used to determine differences among at least three groups. The *p* values for multiple comparisons were adjusted using the Benjamini–Hochberg method. Spearmen or Pearson correlation analysis was conducted to identify and visualize these relationships by applying the “estimate”, “reshape2”, “ggpubr”, “ggplot2”, and “limma” R packages. All linear analysis were performed and visualized using the "impute", "limma", "ggplot2", "ggpubr", "ggExtra", and "corrplot" R packages. In the study, “*”, “**”, and “***” in the research denoted *P* < 0.050 < 0.010 and < 0.001, respectively. A two-sided *P*-value < 0.05 was considered statistically significant. The statistical analyses were performed using R software (R Foundation for Statistical Computing, version 4.0.2, Vienna, Austria, http://www.r-project.org/).

## Supplementary Information

Supplementary Information 1.

Supplementary Information 2.

## Data Availability

The Genotype Tissue Expression (GTEx) and the Cancer Genome Atlas (TCGA) were extracted from the University of California Santa Cruz (UCSC) Xena browser (https://xena.ucsc.edu/). All data supporting the findings of the study are available from the corresponding author on reasonable request. All data in our study are available upon request.

## Abbreviations

GSDMA: Gasdermin A
GSDMB: Gasdermin B
GSDMC: Gasdermin C
GSDMD: Gasdermin D
GSDME: Gasdermin E
PJVK: Pejvakin
GTEx: Genotype-tissue expression
TCGA: The Cancer Genome Atlas
UCSC: University of California Santa Cruz
OS: Overall survival
KM: Kaplan–Meier
ANOVA: Analysis of variance
RNAss: RNA stemness score
DNAss: DNA stemness score
NCI-60: National Cancer Institute-60
ACC: Adrenocortical carcinoma
AML: Acute myeloid leukemia
BLCA: Bladder Urothelial Carcinoma
BRCA: Breast invasive carcinoma
CHOL: Cholangiocarcinoma
DLBC: Lymphoid neoplasm diffuse large B-cell lymphoma
ESCA: Esophageal carcinoma
GBM: Glioblastoma multiforme
HNSC: Head and neck squamous cell carcinoma
KICH: Kidney cancer
KIRC: Kidney renal clear cell carcinoma
KIRP: Kidney renal papillary cell carcinoma
LAML: Acute myeloid leukemia
LGG: Brain lower grade glioma
LIHC: Liver hepatocellular carcinoma
LUAD: Lung adenocarcinoma
LUSC: Lung squamous cell carcinoma
MESO: Mesothelioma
PAAD: Pancreatic cancer endocrine neoplasms
PCP: Pheochromocytoma and paraganglioma
PRAD: Prostate adenocarcinoma
READ: Rectum adenocarcinoma
SARC: Sarcoma
SKCM: Skin cutaneous melanoma
STAD: Stomach adenocarcinoma
TGCT: Testicular germ cell tumor
THCA: Thyroid carcinoma
THYM: Thymoma
UCEC: Uterine corpus endometrial carcinoma
UCS: Uterine carcinosarcoma
UVM: Uveal melanoma
