# Supplementary material for: A comprehensive analysis of gasdermin family gene as therapeutic targets in pan-cancer
Source: Sci Rep. 2022 Aug 3;12:13329. doi: 10.1038/s41598-022-17100-7 (PMC9349317; doi:10.1038/s41598-022-17100-7)
Supplement: Supplementary file 1 — Supplementary Information 1. [file 41598_2022_17100_MOESM1_ESM.docx]

**Supplementary Figure 1:** Kaplan-Meier survival curves comparison of low and high expression of the gasdermin family gene in pan-cancer. GSDMB (A-D), GSDMC (E-K), GSDMD (L-Q), GSDME (R-U), PJVK (V-Z) OS survival curves in different cancers.


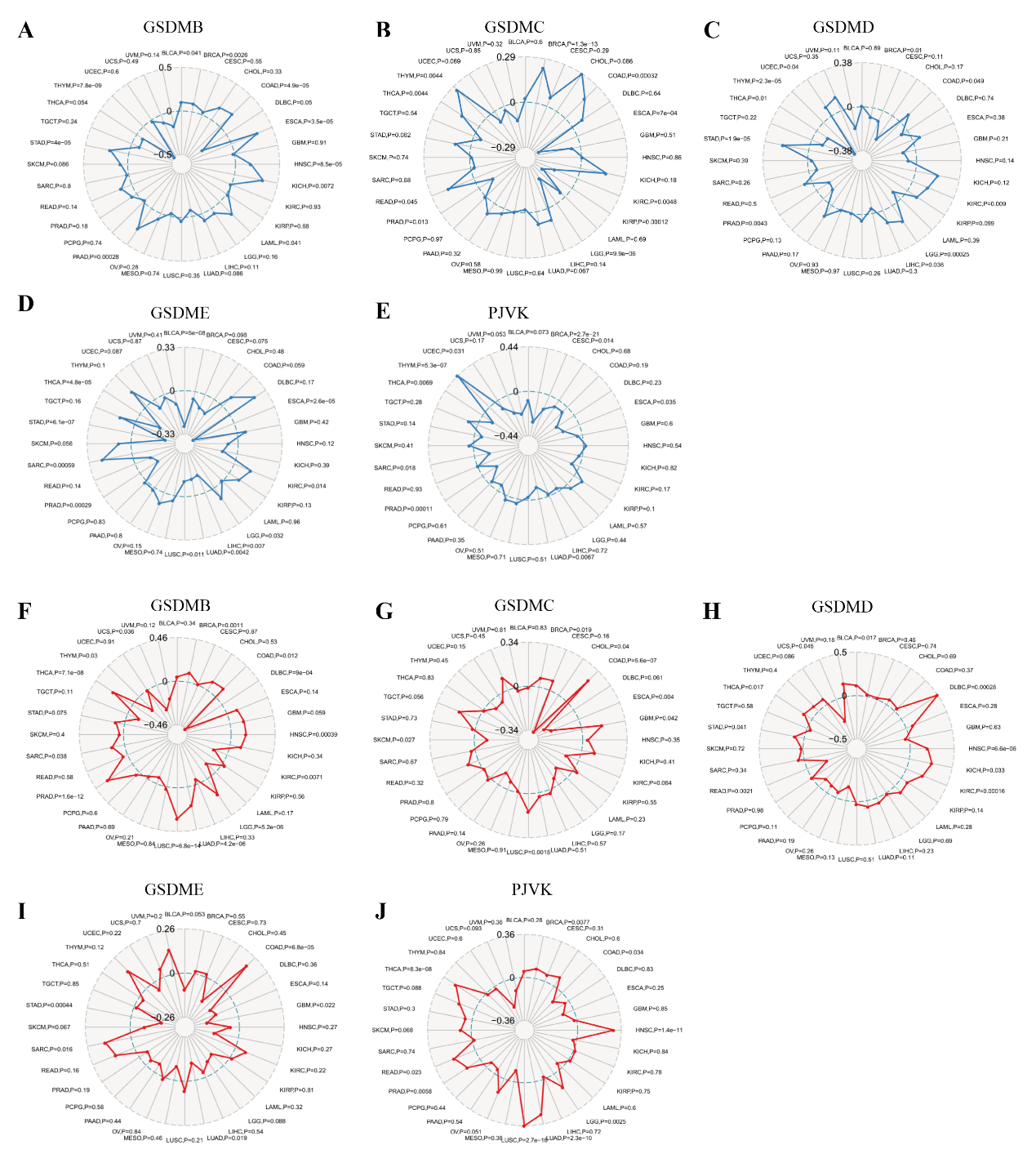


**Supplementary Figure 2:** Correlation analysis of gasdermin family genes expression with tumor mutational burden (TMB) and microsatellite instability (MSI) using Spearman’s rank correlation coefficient. Radar map of correlation between gasdermin family gene expression and TMB; GSDMB(A), GSDMC(B), GSDMD(C), GSDME(D), and PJVK(E). Radar map of correlation between gasdermin family gene expression and MSI; GSDMB(F), GSDMC(G), GSDMD(H), GSDME(I), and PJVK(J).


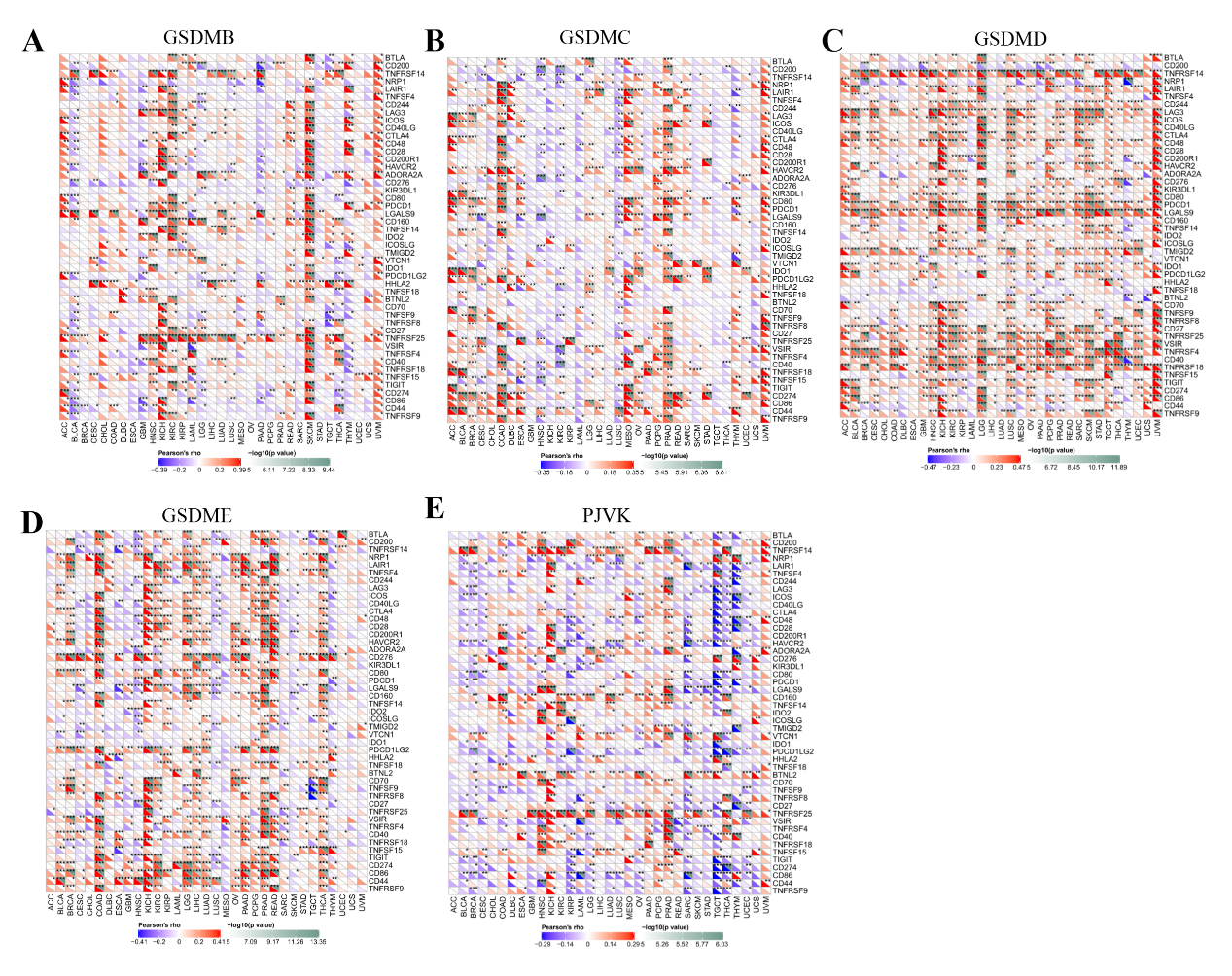


**Supplementary Figure 3:** Correlation analysis of gasdermin family genes expression with 40 common immune checkpoint genes in pan-cancer; The red and blue represented the positive or negative correlation, respectively; *P* < 0.050, *P* < 0.010, and *P* < 0.001were denoted by “*”, “**”, and “***”, respectively. GSDMB(A), GSDMC(B), GSDMD(C), GSDME(D), and PJVK(E).


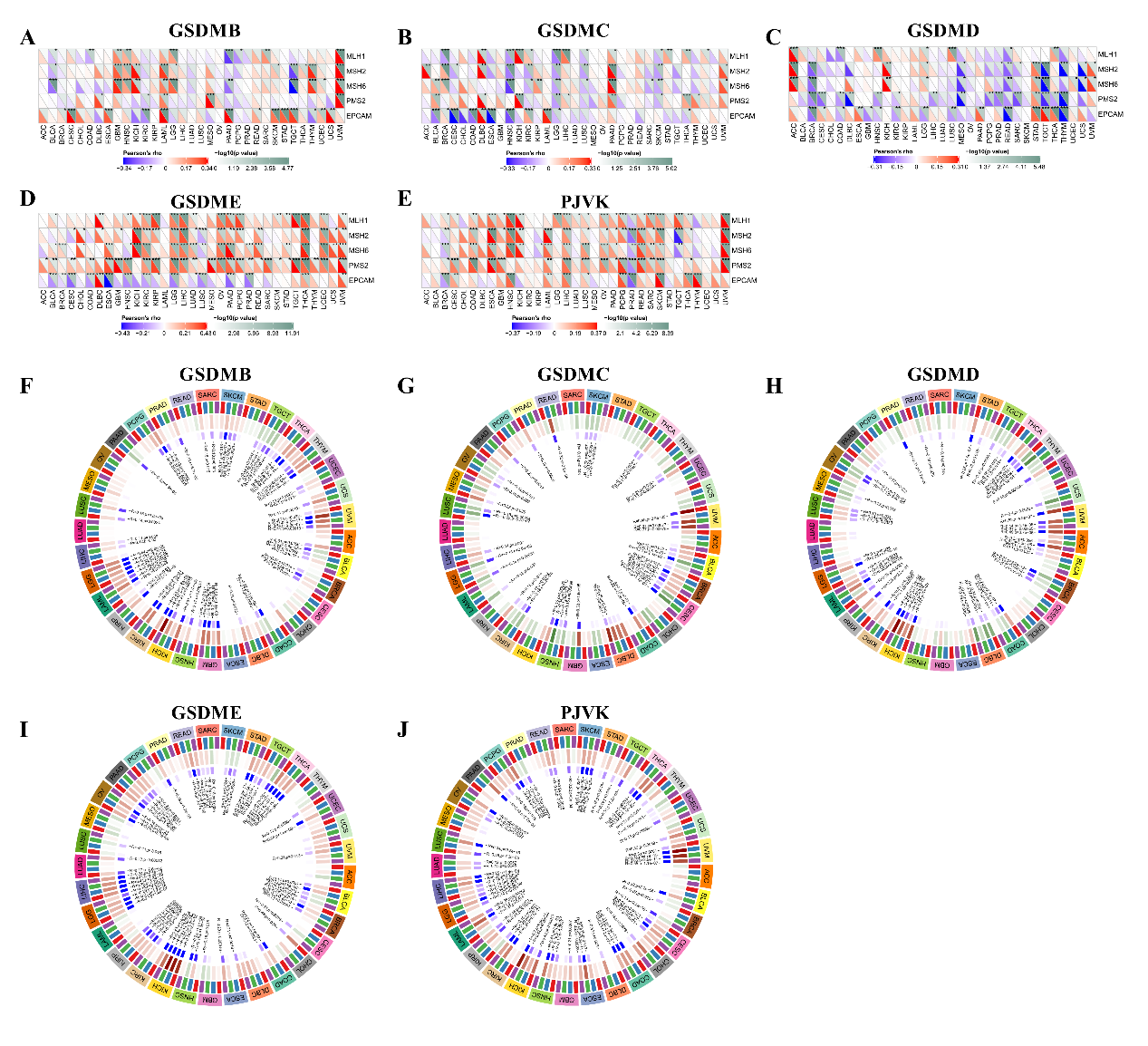


**Supplementary Figure 4:** Correlation analysis of gasdermin family genes expression with the expression levels of DNA mismatch repair (MMR) genes and methyltransferases in pan-cancer. Correlation analysis between gasdermin family gene expression and the expression of the five MMR genes; GSDMB(A), GSDMC(B), GSDMD(C), GSDME(D), and PJVK(E); The red and blue represented the positive or negative correlation, respectively; *P* < 0.050, *P* < 0.010, and *P* < 0.001were denoted by “*”, “**”, and “***”, respectively. Correlation analysis between gasdermin family genes expression and the expression of four methyltransferases; GSDMB(F), GSDMC(G), GSDMD(H), GSDME(I), and PJVK(J); Red, blue, green, and purple represented DNMT1, DNMT2, DNMT3a, or DNMT3b, respectively.


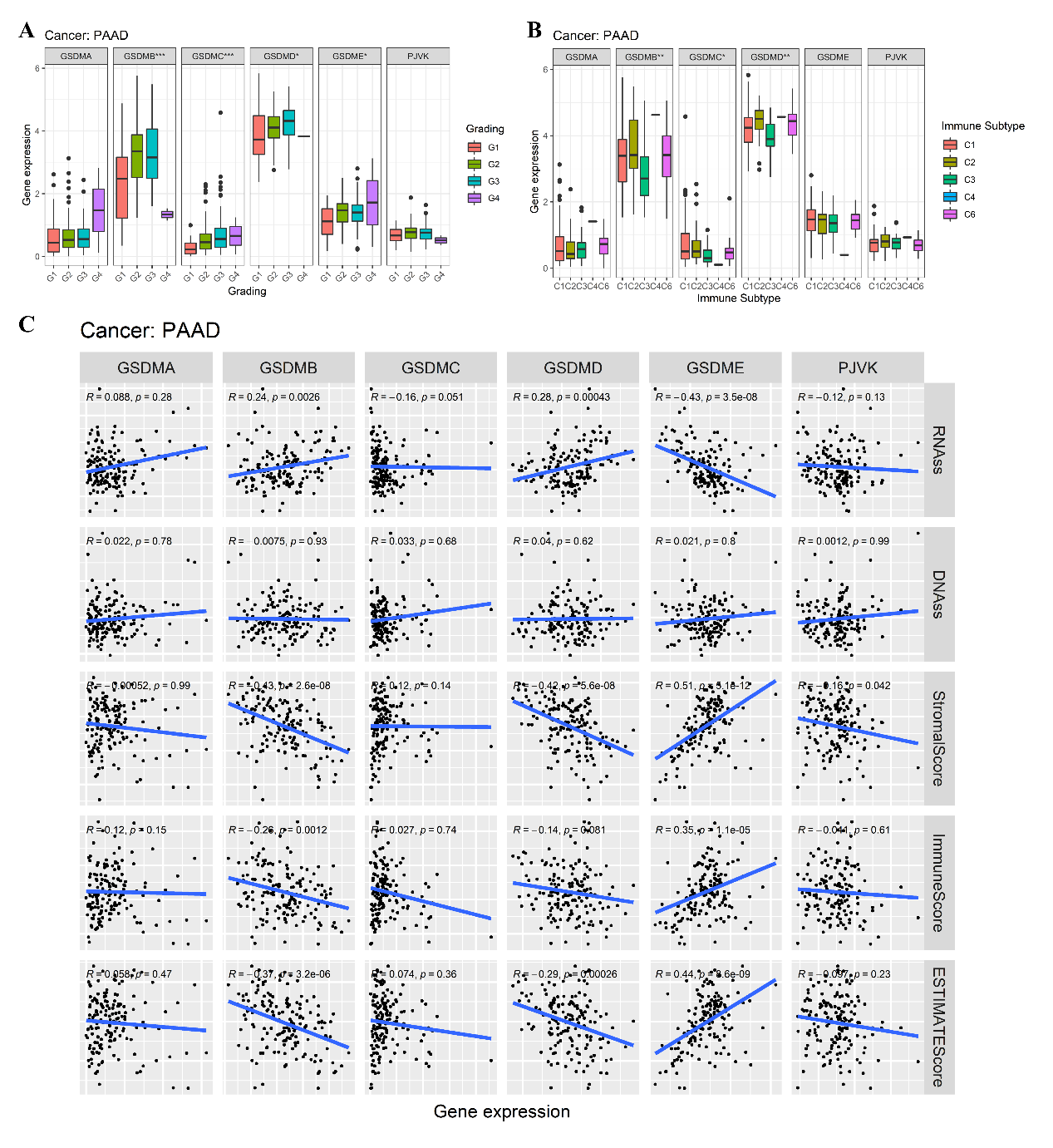


**Supplementary Figure 5:** Associations of gasdermin family gene expression with the clinical grade, immune subtype, stemness score, and tumor microenvironment in PAAD. Gasdermin family genes expression levels in various clinical grades in (A). Associations of gasdermin family gene expression with distinct immune infiltrate subtypes (B). Linear regression analysis showing the correlations of gasdermin family gene expression with stemness score and the tumor microenvironment (C). *P* < 0.050, *P* < 0.010, and *P* < 0.001were denoted by “*”, “**”, and “***”, respectively.


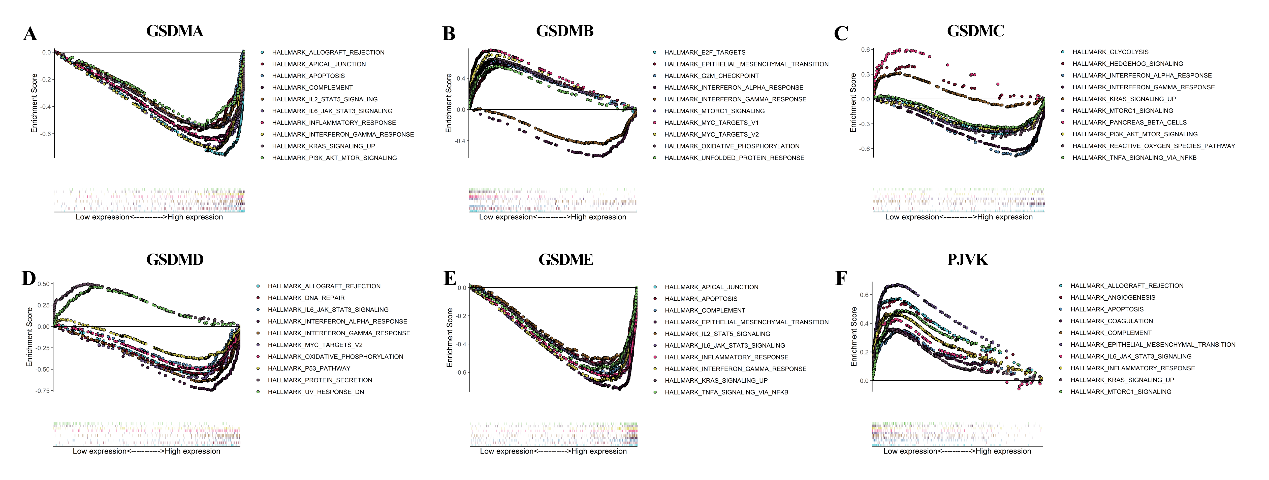


**Supplementary Figure 6:** Gene set enrichment analysis (GSEA) of gasdermin family gene associated with signaling pathways in hallmark dataset. GSDMA (A), GSDMB (B), GSDMC (C), GSDMD (D), GSDME (E), and PJVK (F). Each line representing one particular signaling pathway with unique color, and low-expressed genes lay on the left approaching the origin of the coordinates, and the high-expressed located in the right of X-axis.
