# Supplementary material for: A comprehensive analysis of gasdermin family gene as therapeutic targets in pan-cancer
Source: Sci Rep. 2022 Aug 3;12:13329. doi: 10.1038/s41598-022-17100-7 (PMC9349317; doi:10.1038/s41598-022-17100-7)
Supplement: Supplementary file 2 — Supplementary Information 2. [file 41598_2022_17100_MOESM2_ESM.docx]

**Supplementary Table 1:** Associations of gasdermin family genes expression with immune score

|  | GSDMA | | GSDMB | | GSDMC | | GSDMD | | GSDME | | PJVK | |
| --- | --- | --- | --- | --- | --- | --- | --- | --- | --- | --- | --- | --- |
|  | Correlation coefficient | *P* value | Correlation coefficient | *P* value | Correlation coefficient | *P* value | Correlation coefficient | *P* value | Correlation coefficient | *P* value | Correlation coefficient | *P* value |
| ACC | 0.688 | <0.001 | 0.056 | 0.622 | 0.399 | <0.001 | 0.145 | 0.200 | 0.248 | 0.028 | -0.097 | 0.396 |
| BLCA | 0.085 | 0.084 | -0.208 | <0.001 | 0.317 | <0.001 | 0.391 | <0.001 | 0.198 | <0.001 | -0.238 | <0.001 |
| BRCA | 0.403 | <0.001 | 0.062 | 0.038 | 0.273 | <0.001 | 0.262 | <0.001 | 0.391 | <0.001 | -0.010 | 0.748 |
| CESC | 0.306 | <0.001 | 0.240 | <0.001 | 0.259 | <0.001 | 0.374 | <0.001 | 0.113 | 0.049 | -0.182 | 0.001 |
| CHOL | 0.402 | 0.016 | 0.059 | 0.733 | 0.145 | 0.398 | 0.167 | 0.329 | 0.046 | 0.789 | -0.057 | 0.739 |
| COAD | 0.014 | 0.761 | 0.095 | 0.039 | 0.485 | <0.001 | 0.232 | <0.001 | 0.548 | <0.001 | -0.164 | <0.001 |
| DLBC | 0.135 | 0.359 | 0.170 | 0.247 | 0.147 | 0.318 | 0.177 | 0.229 | 0.278 | 0.056 | -0.193 | 0.188 |
| ESCA | 0.010 | 0.904 | 0.235 | 0.003 | 0.009 | 0.910 | 0.368 | <0.001 | -0.009 | 0.913 | 0.061 | 0.441 |
| GBM | 0.455 | <0.001 | -0.392 | <0.001 | 0.225 | 0.003 | 0.397 | <0.001 | 0.163 | 0.035 | -0.131 | 0.091 |
| HNSC | 0.006 | 0.894 | 0.226 | <0.001 | -0.002 | 0.970 | 0.446 | <0.001 | -0.092 | 0.040 | -0.055 | 0.221 |
| KICH | 0.471 | <0.001 | -0.025 | 0.842 | -0.079 | 0.532 | 0.341 | 0.006 | 0.586 | <0.001 | -0.307 | 0.013 |
| KIRC | 0.464 | <0.001 | 0.228 | <0.001 | 0.060 | 0.164 | 0.292 | <0.001 | 0.395 | <0.001 | -0.027 | 0.527 |
| KIRP | 0.507 | <0.001 | -0.043 | 0.468 | 0.066 | 0.262 | 0.154 | 0.009 | 0.260 | <0.001 | -0.163 | 0.006 |
| LAML | 0.217 | 0.007 | -0.304 | <0.001 | -0.079 | 0.334 | -0.125 | 0.126 | 0.176 | 0.031 | -0.503 | <0.001 |
| LGG | 0.411 | <0.001 | -0.048 | 0.275 | 0.310 | <0.001 | 0.685 | <0.001 | 0.440 | <0.001 | -0.149 | 0.001 |
| LIHC | 0.535 | <0.001 | -0.019 | 0.711 | 0.193 | <0.001 | 0.202 | <0.001 | 0.129 | 0.013 | -0.303 | <0.001 |
| LUAD | 0.344 | <0.001 | 0.116 | 0.008 | 0.008 | 0.846 | 0.197 | <0.001 | 0.364 | <0.001 | -0.096 | 0.028 |
| LUSC | 0.369 | <0.001 | -0.027 | 0.543 | -0.077 | 0.087 | 0.408 | <0.001 | 0.032 | 0.468 | -0.220 | <0.001 |
| MESO | 0.176 | 0.105 | 0.008 | 0.941 | 0.380 | <0.001 | 0.280 | 0.009 | 0.036 | 0.739 | -0.259 | 0.016 |
| OV | 0.428 | <0.001 | 0.104 | 0.043 | 0.453 | <0.001 | 0.349 | <0.001 | 0.213 | <0.001 | -0.210 | <0.001 |
| PAAD | 0.222 | 0.003 | -0.141 | 0.060 | 0.179 | 0.017 | -0.029 | 0.697 | 0.443 | <0.001 | 0.035 | 0.644 |
| PCPG | 0.516 | <0.001 | -0.054 | 0.470 | 0.308 | <0.001 | 0.658 | <0.001 | 0.147 | 0.048 | <0.001 | 0.999 |
| PRAD | 0.196 | <0.001 | 0.130 | 0.004 | 0.453 | <0.001 | 0.328 | <0.001 | 0.557 | <0.001 | 0.235 | <0.001 |
| READ | 0.075 | 0.336 | 0.146 | 0.060 | 0.405 | <0.001 | 0.243 | 0.002 | 0.404 | <0.001 | -0.166 | 0.032 |
| SARC | 0.602 | <0.001 | 0.040 | 0.519 | 0.365 | <0.001 | 0.426 | <0.001 | 0.166 | 0.007 | -0.372 | <0.001 |
| SKCM | 0.379 | <0.001 | 0.329 | <0.001 | 0.248 | <0.001 | 0.437 | <0.001 | 0.018 | 0.703 | -0.110 | 0.017 |
| STAD | 0.219 | <0.001 | 0.078 | 0.132 | 0.147 | 0.004 | 0.164 | 0.001 | 0.135 | 0.009 | -0.121 | 0.019 |
| TGCT | 0.320 | <0.001 | 0.357 | <0.001 | -0.143 | 0.076 | 0.456 | <0.001 | 0.084 | 0.296 | -0.232 | 0.004 |
| THCA | 0.336 | <0.001 | 0.036 | 0.420 | 0.038 | 0.393 | 0.261 | <0.001 | 0.263 | <0.001 | -0.098 | 0.027 |
| THYM | 0.379 | <0.001 | 0.450 | <0.001 | -0.011 | 0.909 | 0.493 | <0.001 | -0.215 | 0.019 | -0.292 | 0.001 |
| UCEC | 0.308 | <0.001 | 0.139 | 0.001 | 0.135 | 0.002 | 0.286 | <0.001 | 0.114 | 0.008 | -0.093 | 0.029 |
| UCS | 0.488 | <0.001 | 0.057 | 0.674 | 0.148 | 0.276 | 0.544 | <0.001 | -0.092 | 0.499 | -0.168 | 0.214 |
| UVM | 0.345 | 0.002 | -0.043 | 0.705 | 0.239 | 0.033 | 0.502 | <0.001 | -0.141 | 0.211 | -0.186 | 0.098 |

**Supplementary Table 2:** Associations of gasdermin family genes expression with stromal score

|  | GSDMA | | GSDMB | | GSDMC | | GSDMD | | GSDME | | PJVK | |
| --- | --- | --- | --- | --- | --- | --- | --- | --- | --- | --- | --- | --- |
|  | Correlation coefficient | *P* value | Correlation coefficient | *P* value | Correlation coefficient | *P* value | Correlation coefficient | *P* value | Correlation coefficient | *P* value | Correlation coefficient | *P* value |
| ACC | 0.546 | <0.001 | 0.064 | 0.575 | 0.354 | 0.001 | 0.201 | 0.076 | 0.135 | 0.237 | -0.173 | 0.128 |
| BLCA | 0.019 | 0.702 | -0.373 | <0.001 | 0.140 | 0.005 | 0.207 | <0.001 | 0.125 | 0.011 | -0.215 | <0.001 |
| BRCA | 0.247 | <0.001 | -0.107 | <0.001 | 0.158 | <0.001 | -0.055 | 0.068 | 0.503 | <0.001 | -0.072 | 0.016 |
| CESC | 0.283 | <0.001 | -0.059 | 0.303 | 0.078 | 0.176 | 0.133 | 0.020 | 0.157 | 0.006 | -0.149 | 0.009 |
| CHOL | 0.546 | 0.001 | -0.073 | 0.672 | 0.268 | 0.114 | 0.016 | 0.927 | 0.003 | 0.987 | -0.043 | 0.804 |
| COAD | -0.026 | 0.575 | -0.226 | <0.001 | 0.503 | <0.001 | -0.018 | 0.700 | 0.844 | <0.001 | -0.051 | 0.268 |
| DLBC | 0.169 | 0.250 | -0.102 | 0.490 | 0.539 | <0.001 | -0.074 | 0.615 | 0.183 | 0.212 | -0.100 | 0.496 |
| ESCA | 0.023 | 0.773 | -0.122 | 0.122 | 0.088 | 0.265 | 0.098 | 0.213 | 0.242 | 0.002 | 0.155 | 0.049 |
| GBM | 0.398 | <0.001 | -0.317 | <0.001 | 0.275 | <0.001 | 0.328 | <0.001 | 0.077 | 0.322 | -0.117 | 0.132 |
| HNSC | 0.062 | 0.166 | -0.187 | <0.001 | -0.096 | 0.031 | 0.101 | 0.023 | 0.057 | 0.206 | -0.130 | 0.004 |
| KICH | 0.459 | <0.001 | -0.131 | 0.299 | -0.093 | 0.459 | 0.200 | 0.110 | 0.659 | <0.001 | -0.237 | 0.057 |
| KIRC | 0.328 | <0.001 | -0.050 | 0.247 | 0.134 | 0.002 | -0.148 | 0.001 | 0.233 | <0.001 | -0.138 | 0.001 |
| KIRP | 0.510 | <0.001 | -0.256 | <0.001 | 0.162 | 0.006 | -0.075 | 0.206 | 0.138 | 0.019 | -0.345 | <0.001 |
| LAML | 0.078 | 0.338 | -0.221 | 0.006 | 0.040 | 0.626 | -0.273 | 0.001 | 0.169 | 0.039 | -0.470 | <0.001 |
| LGG | 0.360 | <0.001 | -0.253 | <0.001 | 0.304 | <0.001 | 0.644 | <0.001 | 0.347 | <0.001 | -0.283 | <0.001 |
| LIHC | 0.416 | <0.001 | -0.227 | <0.001 | 0.203 | <0.001 | -0.100 | 0.053 | 0.028 | 0.583 | -0.284 | <0.001 |
| LUAD | 0.288 | <0.001 | -0.087 | 0.045 | -0.021 | 0.630 | -0.076 | 0.080 | 0.442 | <0.001 | -0.176 | <0.001 |
| LUSC | 0.423 | <0.001 | -0.268 | <0.001 | -0.091 | 0.042 | 0.187 | <0.001 | 0.223 | <0.001 | -0.314 | <0.001 |
| MESO | 0.083 | 0.446 | -0.061 | 0.579 | 0.276 | 0.010 | -0.099 | 0.366 | -0.201 | 0.064 | -0.273 | 0.011 |
| OV | 0.426 | <0.001 | -0.100 | 0.052 | 0.288 | <0.001 | 0.176 | 0.001 | 0.315 | <0.001 | -0.163 | 0.002 |
| PAAD | 0.103 | 0.170 | -0.309 | <0.001 | 0.225 | 0.003 | -0.298 | <0.001 | 0.580 | <0.001 | -0.090 | 0.231 |
| PCPG | 0.466 | <0.001 | 0.032 | 0.670 | 0.386 | <0.001 | 0.758 | 0.000 | 0.082 | 0.268 | -0.168 | 0.023 |
| PRAD | 0.239 | <0.001 | -0.021 | 0.632 | 0.298 | <0.001 | 0.105 | 0.019 | 0.828 | <0.001 | 0.159 | <0.001 |
| READ | 0.037 | 0.636 | -0.167 | 0.031 | 0.467 | <0.001 | 0.041 | 0.597 | 0.724 | <0.001 | -0.087 | 0.262 |
| SARC | 0.609 | <0.001 | -0.107 | 0.084 | 0.266 | <0.001 | 0.127 | 0.040 | 0.142 | 0.021 | -0.393 | <0.001 |
| SKCM | 0.343 | <0.001 | 0.172 | <0.001 | 0.272 | <0.001 | 0.278 | <0.001 | 0.024 | 0.609 | -0.066 | 0.154 |
| STAD | 0.182 | <0.001 | -0.216 | <0.001 | 0.048 | 0.352 | -0.120 | 0.020 | 0.343 | <0.001 | 0.002 | 0.967 |
| TGCT | 0.554 | <0.001 | 0.159 | 0.048 | 0.354 | <0.001 | 0.594 | <0.001 | 0.013 | 0.869 | 0.229 | 0.004 |
| THCA | 0.300 | <0.001 | 0.028 | 0.531 | 0.113 | 0.011 | 0.134 | 0.002 | 0.287 | <0.001 | -0.064 | 0.148 |
| THYM | 0.161 | 0.080 | -0.159 | 0.085 | 0.044 | 0.631 | -0.123 | 0.183 | -0.084 | 0.364 | 0.353 | <0.001 |
| UCEC | 0.318 | <0.001 | 0.007 | 0.865 | 0.007 | 0.861 | 0.024 | 0.571 | 0.370 | <0.001 | -0.005 | 0.901 |
| UCS | 0.357 | 0.007 | -0.127 | 0.351 | 0.072 | 0.597 | 0.097 | 0.478 | -0.165 | 0.224 | 0.054 | 0.690 |
| UVM | 0.267 | 0.016 | -0.080 | 0.481 | 0.115 | 0.310 | 0.359 | 0.001 | -0.159 | 0.160 | -0.222 | 0.048 |

**Supplementary Table 3:** Associations of gasdermin family genes expression with tumor purity

|  | GSDMA | | GSDMB | | GSDMC | | GSDMD | | GSDME | | PJVK | |
| --- | --- | --- | --- | --- | --- | --- | --- | --- | --- | --- | --- | --- |
|  | Correlation coefficient | *P* value | Correlation coefficient | *P* value | Correlation coefficient | *P* value | Correlation coefficient | *P* value | Correlation coefficient | *P* value | Correlation coefficient | *P* value |
| ACC | -0.664 | <0.001 | -0.038 | 0.739 | -0.409 | <0.001 | -0.175 | 0.123 | -0.217 | 0.055 | 0.147 | 0.196 |
| BLCA | -0.050 | 0.314 | 0.314 | <0.001 | -0.239 | <0.001 | -0.316 | <0.001 | -0.166 | 0.001 | 0.237 | <0.001 |
| BRCA | -0.382 | <0.001 | 0.012 | 0.694 | -0.244 | <0.001 | -0.134 | <0.001 | -0.513 | <0.001 | 0.046 | 0.126 |
| CESC | -0.319 | <0.001 | -0.128 | 0.025 | -0.201 | <0.001 | -0.298 | <0.001 | -0.149 | 0.009 | 0.188 | 0.001 |
| CHOL | -0.487 | 0.003 | -0.012 | 0.943 | -0.210 | 0.217 | -0.085 | 0.620 | -0.016 | 0.924 | 0.055 | 0.750 |
| COAD | 0.014 | 0.767 | 0.078 | 0.093 | -0.531 | <0.001 | -0.113 | 0.014 | -0.753 | <0.001 | 0.114 | 0.013 |
| DLBC | -0.128 | 0.384 | -0.040 | 0.789 | -0.446 | 0.002 | -0.004 | 0.980 | -0.280 | 0.054 | 0.188 | 0.200 |
| ESCA | -0.018 | 0.815 | -0.054 | 0.495 | -0.063 | 0.426 | -0.243 | 0.002 | -0.131 | 0.097 | -0.109 | 0.166 |
| GBM | -0.449 | <0.001 | 0.372 | <0.001 | -0.259 | 0.001 | -0.374 | <0.001 | -0.135 | 0.081 | 0.129 | 0.095 |
| HNSC | -0.026 | 0.554 | -0.038 | 0.397 | 0.067 | 0.136 | -0.321 | <0.001 | 0.023 | 0.612 | 0.096 | 0.032 |
| KICH | -0.483 | <0.001 | 0.065 | 0.607 | 0.098 | 0.438 | -0.281 | 0.024 | -0.660 | <0.001 | 0.275 | 0.027 |
| KIRC | -0.462 | <0.001 | -0.128 | 0.003 | -0.113 | 0.009 | -0.116 | 0.024 | -0.372 | <0.001 | 0.086 | 0.046 |
| KIRP | -0.540 | <0.001 | 0.131 | 0.026 | -0.111 | 0.059 | -0.067 | 0.253 | -0.220 | <0.001 | 0.244 | <0.001 |
| LAML | -0.167 | 0.041 | 0.287 | <0.001 | 0.024 | 0.771 | 0.198 | 0.015 | -0.180 | 0.027 | 0.520 | <0.001 |
| LGG | -0.405 | <0.001 | 0.128 | 0.003 | -0.317 | <0.001 | -0.687 | <0.001 | -0.416 | <0.001 | 0.205 | <0.001 |
| LIHC | -0.524 | <0.001 | 0.115 | 0.026 | -0.216 | <0.001 | -0.078 | 0.132 | -0.093 | 0.071 | 0.318 | <0.001 |
| LUAD | -0.347 | <0.001 | -0.020 | 0.653 | 0.008 | 0.850 | -0.067 | 0.125 | -0.436 | <0.001 | 0.148 | 0.001 |
| LUSC | -0.419 | <0.001 | 0.149 | 0.001 | 0.090 | 0.044 | -0.325 | <0.001 | -0.124 | 0.006 | 0.280 | <0.001 |
| MESO | -0.169 | 0.120 | 0.036 | 0.739 | -0.393 | <0.001 | -0.131 | 0.228 | 0.106 | 0.331 | 0.313 | 0.003 |
| OV | -0.465 | <0.001 | -0.004 | 0.938 | -0.414 | <0.001 | -0.296 | <0.001 | -0.294 | <0.001 | 0.210 | <0.001 |
| PAAD | -0.170 | 0.024 | 0.238 | 0.001 | -0.207 | 0.006 | 0.162 | 0.030 | -0.529 | <0.001 | 0.020 | 0.789 |
| PCPG | -0.517 | <0.001 | 0.009 | 0.900 | -0.364 | <0.001 | -0.747 | <0.001 | -0.118 | 0.113 | 0.100 | 0.177 |
| PRAD | -0.239 | <0.001 | -0.064 | 0.151 | -0.407 | <0.001 | -0.243 | <0.001 | -0.736 | <0.001 | -0.216 | <0.001 |
| READ | -0.048 | 0.541 | 0.017 | 0.822 | -0.471 | <0.001 | -0.150 | 0.054 | -0.619 | <0.001 | 0.127 | 0.101 |
| SARC | -0.646 | <0.001 | 0.008 | 0.897 | -0.355 | <0.001 | -0.349 | <0.001 | -0.153 | 0.013 | 0.403 | <0.001 |
| SKCM | -0.393 | <0.001 | -0.293 | <0.001 | -0.279 | <0.001 | -0.402 | <0.001 | -0.019 | 0.683 | 0.094 | 0.042 |
| STAD | -0.218 | <0.001 | 0.078 | 0.131 | -0.106 | 0.040 | -0.019 | 0.709 | -0.257 | <0.001 | 0.064 | 0.214 |
| TGCT | -0.510 | <0.001 | -0.372 | <0.001 | -0.048 | 0.550 | -0.663 | <0.001 | -0.064 | 0.428 | 0.061 | 0.451 |
| THCA | -0.340 | <0.001 | -0.035 | 0.436 | -0.072 | 0.103 | -0.227 | <0.001 | -0.294 | <0.001 | 0.090 | 0.042 |
| THYM | -0.356 | <0.001 | -0.198 | 0.031 | 0.033 | 0.723 | -0.271 | 0.003 | 0.178 | 0.052 | -0.019 | 0.839 |
| UCEC | -0.344 | <0.001 | -0.096 | 0.025 | -0.098 | 0.021 | -0.196 | <0.001 | -0.244 | <0.001 | 0.056 | 0.187 |
| UCS | -0.481 | <0.001 | 0.047 | 0.728 | -0.093 | 0.496 | -0.382 | 0.004 | 0.152 | 0.262 | 0.110 | 0.417 |
| UVM | -0.332 | 0.003 | 0.046 | 0.683 | -0.215 | 0.056 | -0.486 | <0.001 | 0.155 | 0.169 | 0.206 | 0.066 |
